# The Effects of NAA on the Tuberous Root Yield and Quality of *Rehmannia glutinosa* and Its Regulatory Mechanism by Transcriptome and Metabolome Profiling

**DOI:** 10.3390/cimb44080227

**Published:** 2022-07-22

**Authors:** Jianjun Li, Jialin Zhu, Huimin Li, Jingxiao Ma, Peilei Chen, Yanqing Zhou

**Affiliations:** College of Life Sciences, Henan Normal University, Xinxiang 453007, China; 043081@htu.cn (J.L.); 1904183043@stu.htu.edu.cn (J.Z.); 2004283157@stu.htu.edu.cn (H.L.); 2021202@htu.edu.cn (J.M.); chenpl521x@gmail.com (P.C.)

**Keywords:** *Rehmannia glutinosa* Libosch, Naphthylacetic acid, transcriptomics, liquid chromatograph-mass spectrometer, differentially expressed genes, differentially accumulated metabolites

## Abstract

Naphthylacetic acid (NAA) was used to increase the tuberous root yield of *Rehmannia glutinosa*, but the differences between its NAA-treated and control tuberous roots (NT and CG) and the *r*egulatory mechanism of NAA effect remain unclear. In order to investigate them, NTs and CGs were used as materials, and both yield-related indices were measured; the metabolomics and transcriptomics were used to capture differentially accumulated metabolites (DAM) and to validate them via mining differentially expressed genes (DEGs), respectively. The effects of NAA treatment: increased NT mass per plant by 21.14%, through increasing the number of roots and increasing the mean root diameter; increased catalpol content by 1.2234% (*p* < 0.05); up-regulated 11DAMs and 596DEGs; and down-regulated 18 DAMs and 517DEGs. In particular, we discovered that NAA regulated its DAMs and biomass via 10 common metabolic pathways, and that the number of NAA-down-regulated DAMs was more than that of NAA-up-regulated DAMs in its tuberous root. Furthermore, HPLC validated the changes of several DAMs and 15 DEGs (*4CL*, *ARF*, *CCoAOMT*, *ARGOS*, etc.) associated with the yield increase and DAMs were verified by RT-qPCR. This study provided some valuable resources, such as tuberous root indices, key genes, and DAMs of *Rehmannia glutinosa* in response to NAA for distinguishing the CGs from NTs, and novel insights into the **r**egulatory mechanism of NAA effects on both at the transcriptomic and metabolomic levels, so it will lay a theoretical foundation for NAA-regulated plant yield and quality, and provide references for prohibiting the uses of NAA as a swelling agent in medicinal tuber plants in China.

## 1. Introduction

*Rehmannia glutinosa* Libosch (*R. glutinosa*) is a perennial plant with important medicinal and economic values, that is mainly distributed in China, Japan, and Korea. Its tuberous root is used in Chinese traditional medicine, possessing anti-anemic, neuro-protective, anti-inflammatory, hypoglycemic, anti-hypertensive, hepatoprotective, tumor-preventive, and anti-senescence properties [1,2]. Its medicinal properties mainly depend on the yield and quality of its tuberous roots as they contain many bioactive compounds important for this quality [3]. Among them, some primary metabolites contribute to its quality, for example, catalpol and Rehmanioside D as its quality markers in China. Catalpol is an iridoid glycoside with a wide range of pharmacological activities, such as anti-inflammatory and anti-oxidative effects. Rehmanioside D, a carotene glycoside, nourishes Yin, lowers blood sugar, regulates immunity, and tonifies blood. In addition, acteoside was one of its quality markers. On the other hand, its tuberous root yield is related to the mass of tuberous roots per plant, which depend on the root number per plant, root length, and width (thickening) per root [4,5].

The root yield and bioactive metabolites of plants can be affected by both intrinsic and extrinsic factors. Among them, plant growth regulators are widely used in modern agriculture to improve the yield and quality of crops. Of them, auxin is a key regulator of extensive processes of plant growth and development. Its signaling transduction (SCF^TIR1/AFBs^-AUX/IAA-ARF) pathway is a de-repression mechanism of the auxin role, in which IAA is the abbreviation of indole acetic acid. In the SCF^TIR1/AFBs^-AUX/IAA-ARF complex, the SCF (Skp1-cullin-F-box protein) complex is a multiprotein E3 ubiquitin ligase complex, ubiquitylating proteins for their degradation by the proteasome; the auxin response factor (ARF) is a key component of auxin signal transduction. Its disassociation with the auxin/indole-3-acetic acid repressors (Aux/IAAs) or its increased expression can turn on the auxin responsive genes at the downstream of its binding site [6,7]. NAA covers a broad-spectrum, is environmentally friendly, and is a decomposable synthetic auxin [8,9,10]. So far, exogenous NAA has been used to increase plant yield, such as in rice and strawberries [11,12], as well as in changing strawberry metabolites [13]. NAA was also used in *R. glutinosa* tissue culture and genetic transformation for many years, improving *R. glutinosa* rooting or callus and adventitious root induction and shoot differentiation, by itself or in combination with other PGRs [14,15]. Moreover, the effect of a swelling agent (Ligenwei), containing 1% NAA (FoshanYinghui Crop Science Co., Ltd., Foshan, China), on *R. glutinosa* yield was reported [16]. In our previous study, the full-length transcriptome sequencing of NAA-treated *R. glutinosa* grown in its production field was reported [3], but the effects of NAA on the tuberous root yield and quality of *R. glutinosa,* as well as its regulatory mechanism, have not been reported until now.

Secondary metabolites are usually called the end products of gene expression in plants. They can be efficiently identified by untargeted metabolomics, such as liquid chromatograph-mass spectrometer (LC-MS). LC-MS has been widely used to identify multiple metabolites in plants, including *R. glutinosa*, and their accumulation in specific tissues [1]. RNA sequencing is an effective and feasible method for differential gene expression, gene expression profile analysis, and the discovery of novel transcripts in plants [17,18]. Recently, the combination of transcriptome with metabolome has increased the power of bioinformatics analysis, and has been used to explore the important genes participating in the regulatory pathways of specific metabolites for the elucidation of the molecular mechanisms of melatonin’s protective effects in plants [19,20]. However, both integration analysis is not used to investigate the effect of regulatory mechanisms of NAA on the tuberous root yield and quality of *R. glutinosa* until now.

In this study, exogenous NAA was applied to increase the tuberous root yield of *R. glutinosa* in its production field by foliar spray method, its NAA treated and control tuberous roots (NT and CG) were distinguished by measurement and weighing methods, its quality was assessed by HPLC and metabolomics, its transcriptomic and metabolomic changes under NAA were investigated. We discovered some DAMs (especially, down-regulated DAMs) and DEGs, tuberous root indices variations between NT and CG, as well as NAA regulatory mechanisms. This study provided some valuable resources, such as tuberous root indices, key genes, and DAMs of *R. glutinosa* in response to NAA for distinguishing the CGs from NTs, and novel insights into the regulatory mechanism of NAA effects on both at the transcriptomic and metabolomic levels, so it will lay a theoretical foundation for NAA-regulated plant yield and quality, and provide references for prohibiting the uses of NAA as swelling agents in medicinal tuber plants in China. 

## 2. Materials and Methods

### 2.1. Plant Materials

**Figure 1:**
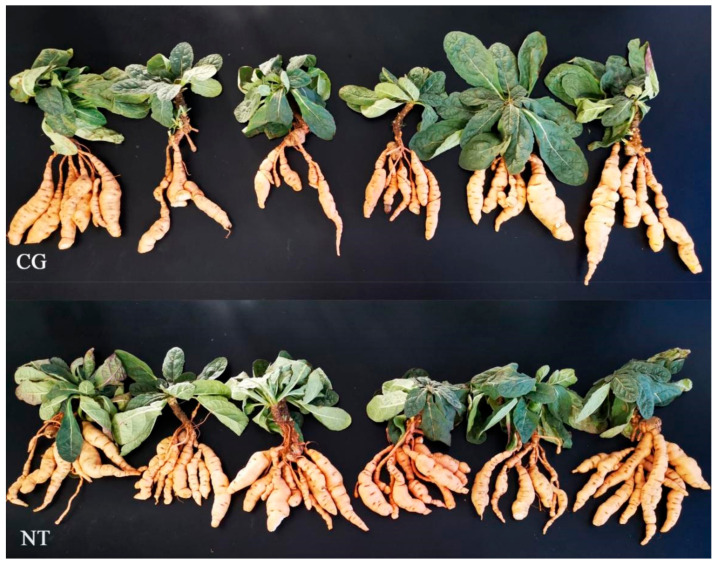
Mature NAA-treated *R. glutinosa* plants and control plants as examples showing their tuberous roots.

Mature plants of *Rehmannia glutinosa* cultivar Jinjiu (MPRs) and its NAA treated plants (NTPs) were provided by Agronomist Cuihong Lu, Wen County Agricultural Science institute, Henan, China, 12 of which were taken for examples (Figure 1). They were all the first crop *Rehmannia glutinosa* prepared according to the report [3] and NAA reagent instructions, with some modifications: the top parts from the fresh and strong roots of *Rehmannia glutinosa* cultivar Jinjiu plants were grown in farmland in Wen County, Henan, China on 20 April 2019. The *Rehmannia glutinosa* farmland was divided into control farmland and treatment farmland, either of which was 666.67 m^2^. More than 10,000 *Rehmannia glutinosa* plants were planted in either farmland. The leaves of those plants in the control farmland were sprayed with water instead of NAA water solution at their elongating and thickening stages. However, the leaves of those plants in the treatment farmland were sprayed with NAA water solution at their elongating and thickening stages according to the following protocol: (1) a mixed solution of 50 mL 5% naphthalene acetic acid (NAA) with 15 L water was sprayed onto the young leaves of young plants on 20 June; (2) a mixed solution of 5 g 99% NAA powder with 15 L water was sprayed onto the young leaves of young plants on 30 June, (3) the same as (1) on the 10 July. When plants were mature, they were harvested on 15 November 2019. Both fresh tuberous roots’ yield was presented in Table S1.

### 2.2. Determination of Main R. glutinosa Root Indices

Then, 6 MPRs and 6 NTPs were randomly selected, whose roots named as NTs and CGs, respectively. Their root indices were analyzed by measurement method, counting, and weighing their mass with ME204 electronic balance (METELER Company, Shanghai, China).

### 2.3. Determination of Catalpol, Rhmannioside D and Acteoside by HPLC

Then, 6 NT samples and 6 CG samples were cut into thin slices and then dried overnight at 55 °C in blast drying oven ZRD-A5110 (Shanghai Zhicheng Analytical Instrument Manufacturing Co., Ltd., Shanghai, China). The dehydrated tuberous root samples were ground into a powder. After being filtered with a 60-mesh sieve, 1 g powder was used for HPLC determination, as described by Li et al. [21]. The reference substances are the following: catalpol (Mans A0215, 20 mg, Purity ≥ 98%), acteoside (Chengdu Mans A0280, 20 mg, Purity ≥ 98%) and Rhmannioside D (Chengdu Mans A1129, 20 mg, Purity ≥ 98%) from Chengdu Must Biotechnology CO.LTD, Chengdu, China.

### 2.4. Transcriptome Sequencing

Then, 3 NT samples and 3 CG samples in dry ice were sent to Biomarker Technologies (Beijing, China) after quick-freezing with liquid nitrogen for next generation sequencing. Total RNA was extracted from freeze-dried fresh tuberous roots using the RNeasy Mini Kit (Beijing equation Jiahong Technology Co., Ltd., Beijing, China) following the manufacturer’s protocol. RNA quality was assessed on an Agilent 2100 Bioanalyzer (Agilent Technologies, Santa Clara, CA, USA) and checked by Rnase-free agarose gel electrophoresis. RNA concentration was determined with Thermo Scientific™ NanoDrop™ 2000/2000c Spectrophotometers. RNA sequencing libraries were constructed with an NEBNext Ultra RNA Library Prep Kit (BioLabs, Inc., Beijing, China) according to the manufacturer’s protocol and sequenced with Illumina Hiseq2500 by Biomarker Technologies (Beijing, China). After the poor quality reads with adaptor sequences, the reads with >10% unknown bases, poly(A)n tails, and low-quality reads (Q value < 20), the clean reads were used for de novo transcriptome assembly by CLC Genomics Workbench (version 6.0.4, CLC Bio., Aarhus, Denmark).

### 2.5. Evaluation of Gene Expression Levels and Data Analyses

Gene expression levels were estimated by fragment per kilo-base per million (FPKM). For each sequenced library, the read counts were adjusted by edge R program package through one scaling normalized factor. Differential expression analysis of two samples was performed using the EBSeq R package [22] from Biomarker Technologies, Beijing, China. The genes with a false discovery rate (FDR) were adjusted using the PPDE (posterior probability of being DE). *P* value (FDR) < 0.01 and |log_2_ Fold Change (FC)| ≥ 1 were considered as the standards for identifying differentially expressed genes (DEGs). The DEGs were annotated to databases such as GO (Gene Ontology), KEGG (Kyoto Encyclopedia of Genes and Genomes), Swissprot (A manually annotated and reviewed protein sequence database), COG (Clusters of Orthologous Groups), Pfam (Protein family), KOG (euKaryotic Ortholog Groups), and NR (NCBI non-redundant protein sequences).

### 2.6. Quantitative Real-Time RT-qPCR

The 3 NT samples and 3 CG samples were cut into thin slices, and then ground into powder in liquid nitrogen. Their powder was used for total RNA extraction and RT-qPCR analysis as stated by Zhou et al. [23]. We selected 15 DEGs related to yield and quality for RT-qPCR analysis using *TIP41* as reference genes [24] to verify the results of transcriptome sequencing and the relationship between these genes and yield or quality. Their primers were designed using the Primer Premier 5.0 (Table S7). Data were presented as a relative transcript level based on the 2^−∆∆Ct^ method [3].

### 2.7. Screening and Identification of Differential Metabolites

The 6 NT samples and 6 CG samples in dry ice were sent to Lianchuan Biotechnology Co., Ltd. (Hangzhou, China) after quick-freezing with liquid nitrogen for metabolome analysis. Metabolites extraction was carried out according to the method [25,26,27]: accurately weigh 50 mg (±1%) of each sample in 2 mL EP tube, and add 0.6 mL of 2-chlorophenylalanine (4 ppm) methanol (−20 °C), vortex for 30 s; add 100 mg glass beads and grind the samples by a high-throughput tissue grinder for 90 s at 60 Hz; ultrasonic treatment for 15 min at room temperature; centrifuge at 4 °C for 10 min at 14,000 rpm, and the supernatant was used as the prepared samples for LC-MS; take 30 µL from each sample to the quality control (QC) samples (These QC samples were used to monitor deviations of the analytical results from these pool mixtures and compare them to the errors caused by the analytical instrument itself) [28]; and, then, use the rest of the samples for LC-MS detection. Chromatographic separation was accomplished in a Shimadzu LC-30A system equipped with an ACQUITY UPLC^®^ HSS T3 (150 × 2.1 mm, 1.8 µm, Waters) column maintained at 40 °C. The temperature of the autosampler was 4 °C. Gradient elution of analytes was carried out with 0.1% formic acid in water (A) and acetonitrile (B) at a flow rate of 0.3 mL/min. Injection of 5 μL of each sample was performed after equilibration. An increasing linear gradient of solvent B (*v*/*v*) was used as follows: 0~0.5 min, 2% B; 0.5~9 min; 2~50% B; 9~12 min, 50~98% B; 12~13 min, 98% B; 13~14 min, 98~2% B; 14~15 min, 2% B [29].

The ESI-MSn experiments were executed on the AB 5600+ mass spectrometer with the spray voltage of 5.50 kV and −4.50 kV in positive and negative modes, respectively. Gas 1 and Gas 2 were both set at 50 psi. Curtain gas was 35 psi. The source temperature was 500 °C. The mass analyzer scanned over a mass range of *m*/*z* 100–1500 for full scan at the collision energy of 45 eV. Dynamic exclusion was implemented to remove some unnecessary information in MS/MS spectra [29]. Through proteowizard software (v3.0.8789), the original data are converted into mzXML format (xcms input file format) [30]; using xcms package of R (v3.3.2) to identify, filter, and align peaks. The main parameters are BW = 5, PPM = 15, peak width = C [5,31], mzwidth = 0.015, mzdiff = 0.01, method = “centWave”; the data matrix, including mass to charge ratio (*m*/*z*), retention time, and peak area, are obtained, in which precursor molecules were obtained in both the positive ion mode and the negative ion mode. The data were exported to Excel for follow-up analysis; To compare the data of different magnitudes, the batch normalization of peak area is carried out. The methods of multivariate statistical analysis (software package Simca-p (v13.0) and R language ropls package) are as follows: principal component analysis (PCA), partial least squares discriminant analysis (PLS-DA), and orthogonal partial least squares discriminant analysis (OPLS-DA) [31]. Based on the screening criteria for differential metabolites: *p*-value ≤ 0.05 and VIP ≥ 1 [32,33], final differential metabolites were screened out. The exact masses of metabolites were confirmed according to mass error < 15 ppm. Then, fragment information from MS/MS mode in data matrix was further matched in databases such as the Human Metabolome Database (HMDB) (http://www.hmdb.ca), Metlin (http://metlin.scripps.edu), massbank (http://www.massbank.jp/), LipidMaps (http://www.lipidmaps.org), mzclound (https://www.mzcloud.org), and the self-built standard product database [34] accessed on 24 March 2020.

### 2.8. Data Analysis

The average value, standard error of the biological replicates, and analysis of variance (ANOVA) were calculated using Microsoft Excel 2010. A *t*-test was served for analysis of significant difference. Single and double asterisks represent the levels of significance at *p*-value < 0.05 and <0.01, respectively.

## 3. Results

### 3.1. Morphological Differences between NTs and CGs and Yield Change

*R. glutinosa* tuberous root weight is vital for its yield, so the NTs from mature NAA-treated *R. glutinosa* plants and the CGs from their control plants were measured and weighed (Table 1 and Table S1). The morphological indices of NT and CG per plant were compared (Table 1), using 6 NT plants and 6 CG plants as materials (Figure 1). NT yield was 39,569.76 kg/ha^2^, which was 13% higher than CG yield (Table S1), suggesting that NAA should improve *R. glutinosa* yield.

### 3.2. DAM Screening and Analyses

LC-MS was used to evaluate the metabolites between CGs and NTs. In total, 83,637 precursor molecules were obtained from CGs and NTs, of which 46,364 were detected in the positive ion mode and 37,273 were detected in the negative ion mode. To further evaluate and identify DAMs, multivariate statistical analyses were performed on the LC-MS data. First, principal component analysis (PCA) was carried out to visualize the metabolite changes in response to NAA treatment in *R. glutinosa* tuberous roots. In PCA score plot (R2 = 0.521) (Figure S1a, see Supplementary Materials), each point represented an independent sample. NT was separated from CG. Then, PLS-DA score plot (R2 = 0.994, Q2 = 0.805) and OPLS-DA score plot (R2 = 0.994, Q2 = 0.676) also showed good separation of both groups (Figure S1b,c). In OPLS-DA score plot (Figure S1c), variable importance in the projection (VIP) is the projection of the importance value of the first principal component variable. Based on the screening conditions: *p*-value ≤ 0.05, VIP ≥ 1, 6125 differential metabolites were filtered from 83,637 precursor molecules, of which 2690 were up-regulated and 3435 were down-regulated in NTs (Figure S2). After that, 29 DAMs were identified by secondary mass spectrometry according to exact mass (The error of measured molecular weight and theoretical molecular weight < 20 ppm) and obtained from their annotated databases, such as Metlin (http://metlin.scripps.edu), MoNA (https://mona.fiehnlab.ucdavis.edu/), and the self-established standard database (Lc-bio Technology Co., Ltd., Hangzhou, China) accessed on 24 March 2020 (Table S2). Among them, 11 metabolites were up-regulated, while 18 metabolites were down-regulated (Figure S3). The first three up-regulated DAMs were kaempferide, *p*-coumaraldehyde, and glyoxylic acid. Two significantly down-regulated DAMs were rosmarinic acid and sinapoyl aldehyde. Based on the relative content of metabolites, their heatmap was constructed (Figure S1d). These 29 DAMs were divided into 7 groups (Figure S4).

### 3.3. Determination of Quality Markers

Among the chemical constituents in *R. glutinosa*, now catalpol and rehmannioside D are its quality markers, and acteoside was ever one of its two quality markers. Therefore, they were determined by HPLC to assess the quality of *R. glutinosa* tuberous roots (Table 2). HPLC analysis indicated that Rehmannioside D and acteoside contents were significantly unchanged between CG and NT, but catalpol content was significantly increased by 1.2234% (*p* < 0.05).

### 3.4. KEGG Metabolic Pathway Analyses of DAMs

**Figure 2:**
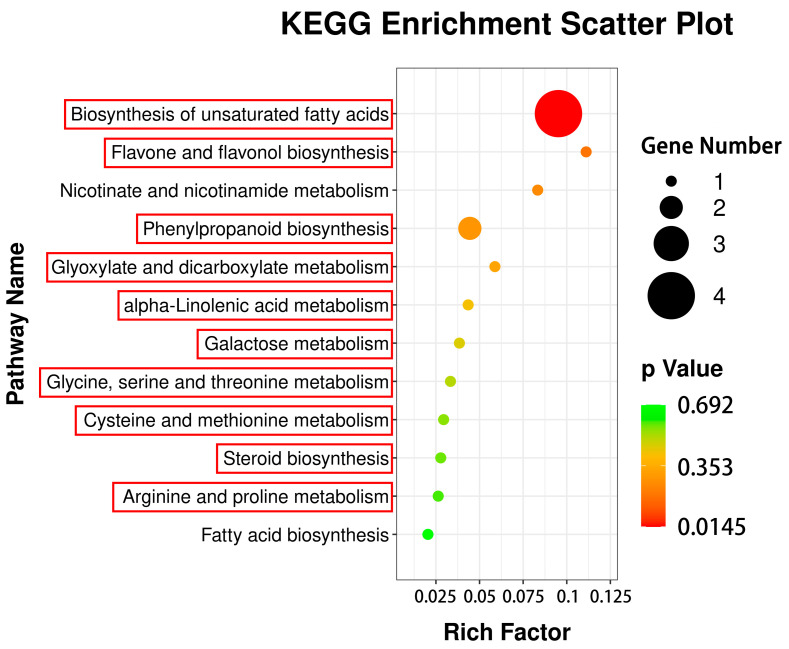
KEGG annotation of DAMs.

**Figure 3:**
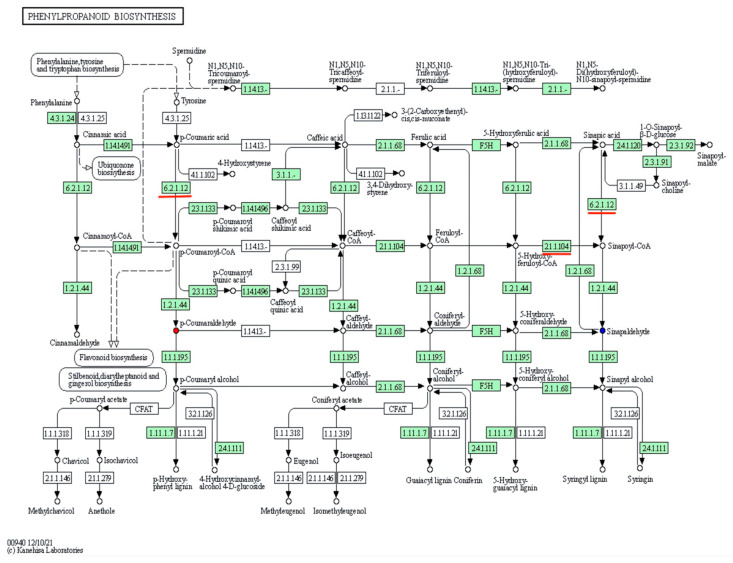
DAM-annotated phenylpropanoid biosynthesis. Red circle: up-regulated DAM. Blue circle: down-regulated DAM. White circle: unchanged metabolite. Arrow: metabolic reaction direction. The number in every box is composed of 4 numbers: EC number. EC numbers in the boxes with green background: enzymes included in *R. glutinosa*. EC numbers in the boxes with white background: enzymes not included in *R. glutinosa*. The numbers underlined in red: the same enzymes as shown in Figure 4.

After 29 DAMs were annotated into KEGG database, it was found that 17 of them were correlated to DEGs in 12 pathways, while others were not annotated into KEGG database (Figure 2). These 17 DAMs are *p*-coumaraldehyde, 3-hydroxybenzoic acid, 3-methylindole, 5,7-dihydroxyflavone, 9(S)-HPOT, 9,10-DHOME, beta-sitosterol, glyoxylic acid, kaempferide, kynurenic acid, lipoxin A4, myristic acid, naringin, prostaglandin-c2, rosmarinic acid, *S*-adenosylmethionine, and sinapoyl aldehyde. Among 12 pathways, phenylpropanoid biosynthesis was illustrated as an example (Figure 3).

Circle size: the size of metabolite number. Circle color: metabolic pathway significance. Red square box: common pathways between DAM- and DEG-annotated KEGG pathways.

**Figure 4:**
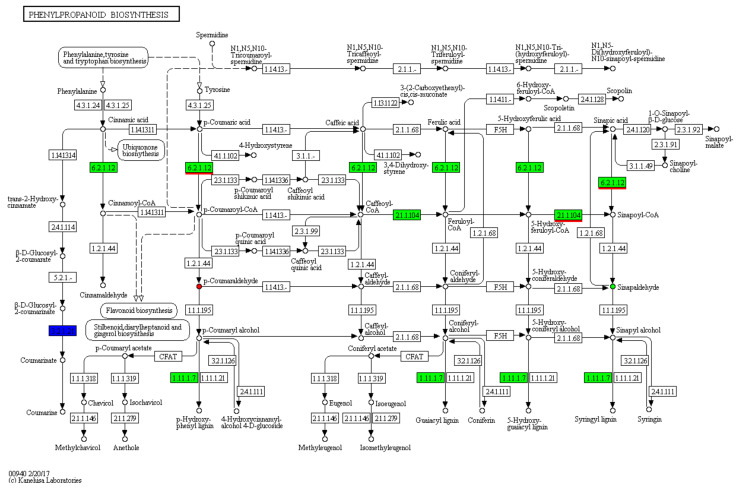
DEG-annotated phenylpropanoid biosynthesis. Red circle: up-regulated DAM. Green circle: down-regulated DAM. White circle: unchanged metabolite. Arrow: metabolic reaction direction. The number in every box composed of 4 numbers: EC number. EC numbers in the boxes with green background: enzymes included in *R. glutinosa.* EC numbers in the boxes with white background: enzymes not included in *R. glutinosa.* EC numbers in the boxes with white background: enzymes not included in *R. glutinosa.* EC numbers in the blue box: enzymes included, up- and down-regulated in *R. glutinosa.* 4-coumarate-CoA ligase (EC 6.2.1.12), caffeoyl-CoA O-methyltransferase (EC 2.1.1.104), and peroxidase (EC1.11.1.7) in green boxes. beta-glucosidase (EC3.2.1.21) in the blue box. The numbers underlined in red: the same enzymes as shown in Figure 3.

### 3.5. Identifying DEGs by Transcriptome Sequencing and Their Function Classifications

**Figure 5:**
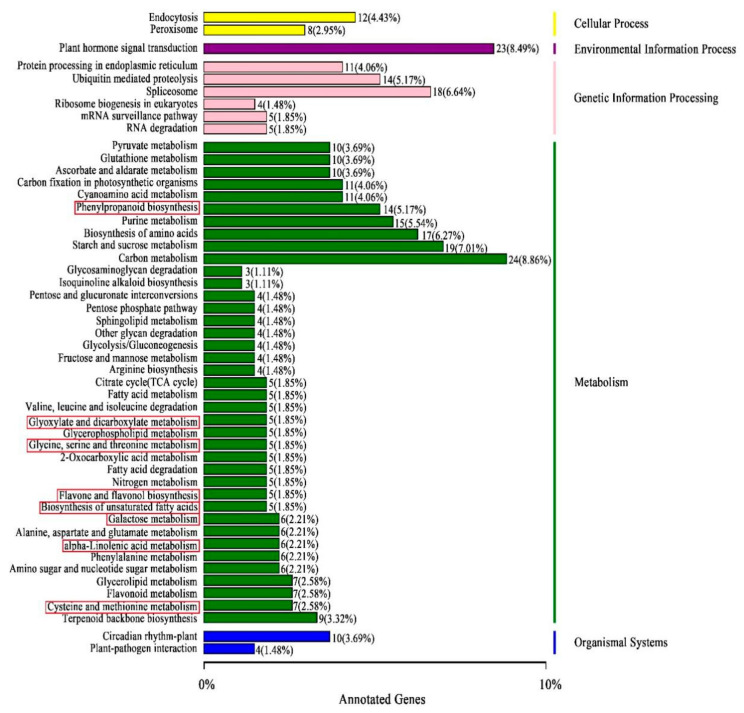
KEGG pathway analyses of DEGs showing the first 50 pathways.

To further explore the regulatory mechanism of NAA effects on these DAMs and yield, we performed transcriptome sequencing of the NT and CG samples. Its data indicated that their GC contents were all more than 44%, and the average base mass of Q30 was more than 93%, suggesting that it was of good quality and met the criteria of further data analysis. The clean reads from NT and CG samples were obtained after the low-quality reads were filtered (Tables S2 and S3). According to |log2 FC| ≥ 2 and false discovery rate (FDR) < 0.01, a total of 1113 DEGs were identified between NTs and CGs, of which 596 were up-regulated and 517 were down-regulated (Figure S5). These DEGs were described in eight databases, such as GO and KEGG pathway analyses, to identify the genes associated with NAA effects and regulation in *R. glutinosa* (Table S4, Figure 5 and Figure S6). Figure S6 showed that the DEGs were classified into molecular function, biological process, and cellular component. Those DEGs related to the metabolic process; developmental process; and response to stimuli, growth, and signal would be associated with yield and metabolism and further studied.

Red square box: common pathways between DAM- and DEG-annotated KEGG pathways. Number and percentage: the number of the DEGs annotated to each KEGG pathway and (the number of the DEGs annotated to each KEGG pathway: the number of all DEGs annotated to all KEGG pathways) × 100%.

### 3.6. Correlation Analysis of DEGs to DAMs via Common KEGG Pathways

In total, 454 DEGs were annotated to 127 KEGG pathways, 50 of which were significantly enriched pathways with the lowest Q-value (Figure 5). According to their types, they were classified into five groups: cellular process (20, 7.38%), environmental information process (plant hormone signal transduction) (23, 8.49%), genetic information processing (57, 21.07%), metabolism (156, 57.89%), and organismal systems (14, 5.17%). In metabolism, there were 39 metabolic pathways (Figure 5), 8 of which were the same as that in 12 DAM-annotated metabolic pathways (Figure 2). These 8 pathways were boxed in red (Figure 2 and Figure 5), including: phenylpropanoid biosynthesis (Figure 4); glyoxylate and dicarboxylate metabolism; glycine, serine, and threonine metabolism; flavone and flavonol biosynthesis; biosynthesis of unsaturated fatty acids; galactose metabolism; alpha-linolenic acid metabolism; and cysteine and methionine metabolism. In fact, if the lowest Q-value was enlarged, steroid biosynthesis and arginine and proline metabolism of the 12 DAM-annotated pathways could be also found in the 127 KEGG pathways (Figure S7). Therefore, there were 10 common pathways between the 12 pathways (Figure 2) and the 127 pathways (Figure 5 and Figure S7). Based on correlation coefficients, some DEGs were correlated to DAMs via common KEGG pathways (Table S5).

### 3.7. Regulation of These DEGs on These DAMs

**Figure 6:**
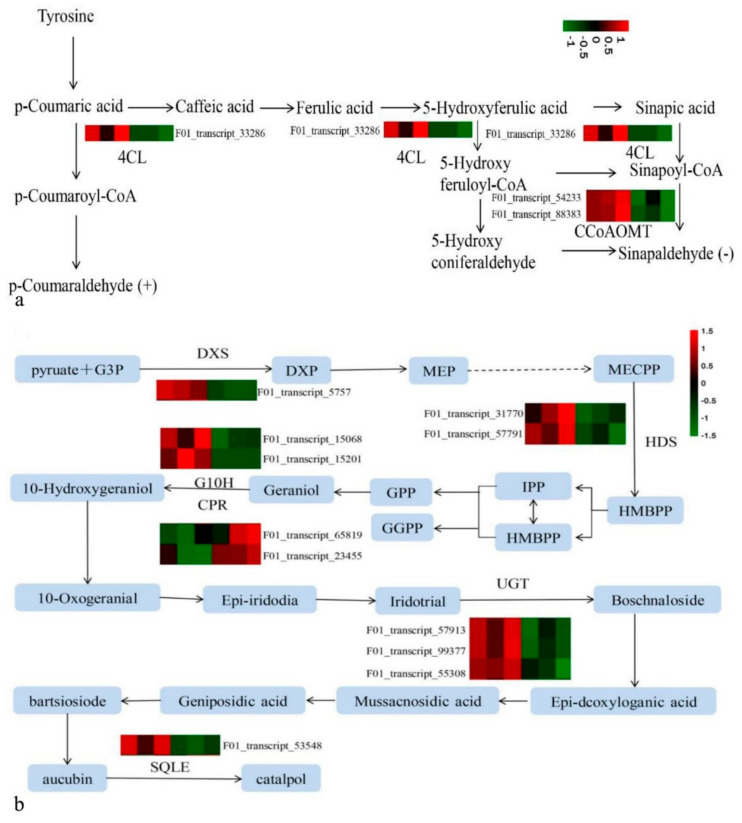
Regulation of DEGs on DAMs via metabolic pathways. (**a**) Regulation of DEGs on DAMs (*p*-coumaraldehyde and sinapoyl aldehyde) in Phenylpropanoid biosynthesis (Figure S8). +: up-regulated. −: down-regulated. 4CL: 4-coumarate-CoA ligase (F01_transcript_33286) (EC 6.2.1.12). CCoAOMT: caffeoyl-CoA O-methyltransferase (F01_transcript_54233, F01_transcript_88383) (EC 2.1.1.104). (**b**) Regulation of DEGs on catalpol in its biosynthesis pathway. DXS: 1-deoxy-D-xylulose-5-phosphate synthase (F01_transcript_5757). HDS: 4-hydroxy-3-methylbut-2-en-1-yl diphosphate synthase (F01_transcript_57791, F01_transcript_31770). G10H: Geraniol 8-hydroxylase (F01_transcript_15068, F01_transcript_15201). UGT: UDP-glucosyltransferase (F01_transcript_57913. F01_transcript_99377, F01_transcript_55308). SQM: Squalene monooxygenase (F01_transcript_53548). CPR: cytochrome P450 reductase (F01_transcript_23455, F01_transcript_65819). Heat maps: Differential expressions of DEGs between CGs (CG_1_-CG_3_) and NTs (NT_1_-NT_3_).

Based on the phenylpropanoid biosynthesis (Figure 3) and the phenylpropanoid biosynthesis (Figure 4) in Section 3.6, phenylpropanoid biosynthesis was taken as an explanation of the regulation of some DEGs on two DAMs; in other words, it was expression analysis of differential genes related to two DAMs, such as *p*-coumaral and sinapoyl aldehyde in the KEGG metabolic pathway (Figure 6a). In Figure 4, Beta-glucosidase1 gene (*BGLU1*) was up-regulated, while 12 genes were down-regulated, including Peroxidase 21 gene (*PER21*), 4-coumarate-CoA ligase (EC 6.2.1.12) gene (*4CL*), caffeoyl-CoA O-methyltransferase (EC 2.1.1.104) gene (*CCoAOMT*), and Raucaffricine-O-beta-D-glucosidase gene (*RG*).

Their cooperation up-regulated *p*-coumaraldehyde and down-regulated sinapoyl aldehyde. Both comparative analyses unveiled that there were two common DAMs (*p*-coumaral and sinapoyl aldehyde) and two common DEGs (*4CL* and *CCoAOMT*) (Figure 3 and Figure 4) in those metabolic steps at the upstream of both DAMs, whose encoding enzyme numbers are EC 6.2.1.12 and EC 2.1.1.104.

In addition, catalpol belongs to iridoid glucosides, which are synthesized via MEP and MVA pathways [17]. The complete pathway of catalpol biosynthesis has not been fully elucidated and deposited in the KEGG database up to date, so we used the catalpol biosynthesis pathway to elucidate its regulation [17]. In this pathway, 11 candidate DEGs were screened out, which encode 6 enzymes (Figure 6b). The 2 genes involved in catalpol synthesis pathway were up-regulated, including cytochrome P450 reductase (*CPR*), while 9 genes involved in catalpol synthesis pathway were down-regulated, including 1-deoxy-D-xylulose-5-phosphate synthase (*DXS*), 4-hydroxy-3-methylbut-2-en-1-yl diphosphate synthase (*HDS*), geraniol 8-hydroxylase (*G10H*), UDP-glucosyltransferase (*UGT*), and squalene monooxygenase (*SQLE*).

### 3.8. Regulation of DAMs by Transcription Factors

In total, 5562 transcription factors (TFs) were predicted from NGS data by iTAK [3], which were divided into 20 types [3]. Based on correlation coefficients, 96 TFs correlated to DAMs were identified (Table S6). Among them, 55 TFs and DAMs were regulated by NAA in the same up-regulation way or down-regulation way, while 41 TFs and DAMs in the opposite way. On the other hand, some TFs could be identified from those 23 DEGs annotated to plant hormone signal transduction (Table 3). Among them, F01_transcript_42053, F01_transcript_88073, and F01_transcript_9341 encode ethylene-insensitive protein3; F01_transcript_13026 and F01_transcript_13157 encode BR-signaling kinase; F01_transcript_4394 and F01_transcript_4877 encode EIN3-binding F-box protein; F01_transcript_46845 encode serine/threonine-protein kinase CTR1; F01_transcript_54031, F01_transcript_16804, and F01_transcript_17041 encode brassinosteroid resistant 1/2; F01_transcript_60137, F01_transcript_10164, and F01_transcript_40403 encode protein phosphatase 2C; F01_transcript_2057 and F01_transcript_36141 encode the ethylene receptor; F01_transcript_62494 encode cyclin D3; F01_transcript_16175 encode serine/threonine-protein kinase SAPK2; F01_transcript_73250 encode jasmonate ZIM domain-containing protein; F01_transcript_21517 encode auxin-responsive protein IAA; and F01_transcript_29473 encode the abscisic acid receptor PYR/PYL family. For example, 5,7-dihydroxyflavone was regulated by 25 DEGs, including EIN3 (TFs) (Table 3).

### 3.9. Identification of the DEGs Associated with Yield

Among those DEGs regulating DAMs (Table 4), RFS, 4CL, BGLU, and CCoAOMT could also regulate the tuberous root yield according to the previous reports [18,19,20,35]. Except for these four DEGs, other five DEGs, regulating the root yield, were also identified by their descriptions in the Uniprot database (https://www.uniprot.org/, accessed on 24 March 2020) to check their functions (Table 4), whose names and functions were described in the previous reports [36,37,38,39,40,41,42,43,44]. These DEGs improved the tuberous root yield of *R. glutinosa* through increasing its length, biomass, branching number, and width (diameter or thickening).

### 3.10. Verification of DEGs Related to Quality and Yield

**Figure 7:**
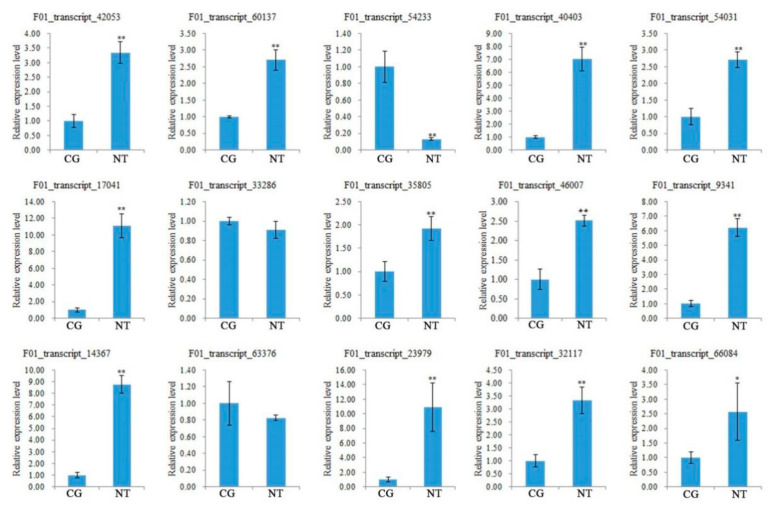
RT-qPCR analyses of DEGs related to *R. glutinosa* tuberous root quality and yield. *, ** indicate significance at *p* ≤ 0.05, *p* ≤ 0.01, respectively. F01_transcript_42053 and F01_transcript_9341 (Protein ethylene insensitive3), F01_transcript_60137 and F01_transcript_40403 (Protein phosphatase 2C), F01_transcript_54031 and F01_transcript_17041 (Protein BZR1 homolog1), F01_transcript_33286 (4-coumarate-CoA ligase 2), F01_transcript_46007 and F01_transcript_35805 (Auxin-regulated gene involved in organ size), F01_transcript_54233 (Caffeoyl-CoA O-methyltransferase), F01_transcript_14367 (Beta-glucosidase), F01_transcript_63376 (lipoxygenase), F01_transcript_23979 (F-box protein GID2), and F01_transcript_32117 and F01_transcript_66084 (Allantoinase).

To verify the DEGs mined by the transcriptome sequencing and their correlation to NT quality and yield, 15 DEGs related to both quality and yield were selected from those DEGs related to both quality and yield in the above data (Table 3 and Table 4), of which 12 up-regulated DEGs and 3 down-regulated DEGs were verified by RT-qPCR using *TIP41* as the reference gene (Table S7). Total RNAs as templates each were extracted from NTs or CGs per *R. glutinosa* plant. *TIP41* expression level was set as 1 for every DEG gene, while every DEG gene expression level was its relative value (Figure 7). It was seen that 12 up-regulated DEGs mined by NGS were all up-regulated, while 3 down-regulated ones were all down-regulated in high-yield NTs. These results indicated that both detection results were consistent with each other, and that these DEGs were indeed related to yield and quality.

### 3.11. Identification of Auxin Regulatory Factors and NAA Regulatory Mechanism

Based on the de-repression mechanism of auxin role [6,7], some NAA regulatory factors, such as ARFs, Aux/IAAs, TIR1, and SCFs were identified from the DEGs (Table 5). Among them, SCF (Skp1-cullin-F-box protein) complex is a multiprotein E3 ubiquitin ligase complex, ubiquitylating proteins for their degradation by the proteasome. It was seen from Table (1) that 1 AUX/IAA gene, 12 SCF genes, and an ARF gene (F01_transcript_46036) were down-regulated, while TIR1s were normal, and (2) that the other ARF gene (F01_transcript_77495) were up-regulated. The result (1) suggested that the NAA regulatory mechanism was consistent with the de-repression mechanism of auxin role, in which bound ARF repressed its downstream DEGs, but the result (2) suggested that ARF gene (F01_transcript_77495) had a different function from and ARF gene (F01_transcript_46036), whose up-regulation promoted its downstream DEGs. As a result, both ARF genes led to the up or down of those DAMs.

To sum up, the regulation mechanism of NAA-improving yield and NAA-affecting metabolites of *R. glutinosa* is the following (Figure 7).

## 4. Discussions

### 4.1. NAA Could Increase the Yield Related Indices of R. glutinosa Tuberous Roots

*R. glutinosa* is a kind of herb with tuberous root as medicine. Its tuberous root weight is a major standard of its yield. In this study, NAA could increase the yield of *R. glutinosa* by about 13% measured by Agronomist Lu of Wen County Institute of Agricultural Sciences and its field growers (Table S1). Her previous report exhibited the improvement of a swelling agent (Ligenwei, Foshan Yinghui Crop Science Co., Ltd., Foshan, China), containing 1% NAA, in its yield [16]. However, the molecular regulatory mechanism of NAA effect on its tuberous root has not been reported until now. In this study, we discovered the increase in its tuberous root yield through increasing root number, mass, and mean root diameter per plant (Figure 1 and Table 1). After intensively studying the yield increased DEGs, we obtained some DEGs related to it, for instance, auxin-regulated gene involved in organ size (*ARGOS*, F01_transcript_35805, and F01_transcript_46007), protein phosphatase 2C, brassinosteroid restant 1/2, MADS-box, ARFs, and so on (Table 4). It is generally believed that the root expansion is mainly affected by environmental factors, changes of assimilation products, endogenous hormones during root enlargement, and genes [45]. The previous studies demonstrated that the changes of enzymes and gene expression significantly affected plant root enlargement [46]; for example, the changes of genes related to lignin and starch synthesis and metabolism significantly affected rice and tuber crops’ root enlargement [47,48,49]; NAA as a synthetic auxin could also effectively improve their qualities and increase their economic benefits [47,48,49]. During the formation of Gladiolus bulbs, LOX could initiate the synthesis of oxidized lipid regulating cell growth, and control the growth and development of tubers [43]. It was found that some DEGs, such as *AUX/IAA*, *TIRI*, *ARFs,* and *CH3*, were matched to the gene families related to plant signal transduction pathways in the study of radish, which were involved in tryptophan metabolism, zeatin synthesis, and brassinolide synthesis. These transcripts could play a positive role in the expansion of fleshy roots by affecting cell division and expansion [50]. The genes related to rhizome enlargement of lotus root, including the hormone-inducible protein gene, light-inducible protein (MADS-BOX) gene, rhizome storage protein gene (*Patatin*), starch metabolism related genes, and rhizome formation related genes, could enlarge its roots [44,51]. Some *ARGOS* genes, affecting plant organ size, have been isolated [39,52]. These results will have important reference value for understanding the regulatory mechanism of NAA effect on the root yield of *R*. *glutinosa* and provide functional genes for genetic improvement of *R*. *glutinosa*.

### 4.2. NAA Changed the Quality of R. glutinosa

Secondary metabolites in *R. glutinosa* are vital for its quality, now whose index components are catalpol and Rehmannioside D [3]. Both contents are important indexes for its quality control. HPLC analysis unveiled that catalpol content was obviously increased, and Rehmannioside D content was slightly increased by NAA in NTs compared with that in CGs (Table 2), suggesting that NAA treatment increases its NT quality. However, our metabolome analysis indicated that there were 29 DAMs between CG and NT, of which 11 were up-regulated, while 18 were down-regulated (Table S2). This result indicated that the number of NAA-down-regulated DEGs was more than that of NAA-up-regulated DEGs, suggesting a certain negative effect of NAA treatment on its quality. Before this study, a report showed that NAA treatment could change the contents of anthocyanins, polyphenols, vitamin C, and other substances in blueberries, suggesting the improvement of NAA in ripe blueberry fruit quality to some extent [53]. Another report suggested that NAA could not only increase the contents of some metabolites, but also decrease that of other metabolites in *Vitis vinifera* [54]. What is more, in other plant species, NAA could also effectively improve their qualities and increase their economic benefits [51,55,56]. Therefore, our present results are similar to these reports mentioned above. One of them [54] is consistent with one of our results that NAA could not only down-regulate some DEGs but also up-regulated other DEGs. However, we discovered that the number of NAA-down-regulated DAMs was more than that of NAA-up-regulated DAMs in *R. glutinosa* tuberous root (Table S2).

### 4.3. Transcriptomics Analysis Validated the Effect of NAA on R. glutinosa Tuberous Root Quality

After metabolomics identified 29 DAMs, our transcriptomics validated the genes related to some of them based on the correlation coefficient (Table 3 and Tables S5 and S6), which encodes some enzymes catalyzing their metabolic pathways. In addition, there were 10 common KEGG pathways between the DEG-annotated KEGG pathways and DAM-annotated KEGG pathways. Among these 29 DAMs, 17 were involved in these 10 common KEGG pathways (Figure 2 and Figure 5). Among these results, the one that DAMs were involved in many KEGG pathways is consistent with the previous report [13]. Moreover, RT-qPCR detection validated the transcriptome sequencing data’ accuracy, and verified the relation of the expression levels of some DEGs, associated with yield and quality, to the yield indices per plant, and some DAM between the NTs and the CGs (Figure 7).

### 4.4. Molecular Regulatory Mechanism of NAA Effects on R. glutinosa Quality and Yield

Our metabolomics results indicated that NAA mainly affected 12 crucial metabolic pathways, such as biosynthesis of unsaturated fatty acids, phenylpropanoid biosynthesis, flavone and flavonol biosynthesis, and so on (Figure 2), suggesting that it was not likely to play a role via a specific enzyme but via an enzyme cascade. Transcriptomics analysis validated the variation between the CG and the NT, and the crucial genes of some DEMs related to 10 common metabolic pathways were verified by RT-qPCR (Figure 7 and Figure S7). Therefore, our metabolomics analysis was consistent with transcriptomics analysis to a large extent. Meanwhile, both analyses also showed that NAA mainly affected the biomass in the NT (Table 4). On the other hand, some DEGs related to the tuberous yield indices were screened based on the previous reports (Table 4) and confirmed by RT-qPCR-based correlation analyses of their expressions to yield indices between CG and NT, for example, between the CG and a NT with NAA-enlarged roots. These results laid the foundation for identifying differential metabolites responsible for NAA effects and further investigating the relevant regulatory mechanisms.

**Figure 8:**
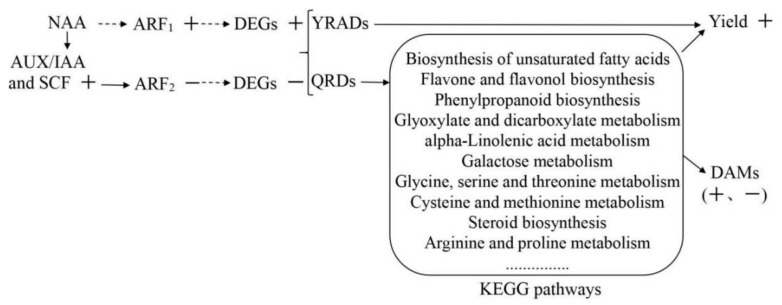
Regulation mechanism of NAA on the tuberous root metabolite and yield of *R. glutinosa*. +: up-regulated. −: down-regulated. YIRD: yield-related DEG. QRD: quality-related DEG. Virtual arrow: NAA and ARFs function via the intermediate molecule(s) unidentified by this study……: more metabolic pathways.

However, how NAA regulated the DEGs in its regulatory mechanisms should be further explained. It was explained as follows: NAA belongs to auxin, so its regulation mechanism is similar to that of auxin (IAA). Auxin plays a very important role in plant growth and development. According to the de-repression mechanism of auxin role [6,7], when the concentration of auxin is low, the AUX/IAA and SCF^TIR1^ complex binds to the auxin response factor (ARF) activator to turn off auxin responsive genes, so their transcriptions are repressed. When the concentration of auxin increases, the AUX/IAA and SCF^TIR1^ complex are degraded by 26S protease so that the ARFs were released from the SCF^TIR1_^AUX/IAA-ARF complex and activate the expression of auxin-responsive genes [13,57,58]. In this study, NAA treatment did not change TIR1level, but it up-regulated the levels of AUX/IAA, SCF^TIR1^ and one ARF (F01_transcript_79445), while it down-regulated the other ARF (F01_transcript_46036) (Table 5). As a result, both ARF genes were regulated by exogenous NAA in different ways. Based on the de-repression mechanism of the auxin role [6,7], F01_transcript_46036 could be down-regulated by up-regulated AUX/IAA and SCF^TIR1^ so that some NAA-responsive DEGs were down-regulated (Figure S5), and then down-regulated some DAMs (Figure 4). On the other hand, F01_transcript_79445 was up-regulated by means of the other function different from F01_transcript_46036 and then turned on other NAA-responsive genes-DEGs (Figure 3, Figure 4 and Figure S5). Some ARFs, such as F01_transcript_79445, were reported [6]. Among them, some DEGs just controlled the biosyntheses of some DAMs via metabolic pathways, for example, catalpol content (Figure 4) and beta-sitosterol, *S*-adenosylmethionine, 3-hydroxybenzoic acid, Naringin, Glyoxylic acid, Sinapoyl aldehyde, 5,7-dihydroxyflavone, Kaempferide (Table 3); other DEGs could not only regulate DAMs but also tuberous root yield via metabolic pathways, e.g., RFS, 4CL, BGLU, and CCoAOMT (Table 3); still other DEGs could only increase NT yield, for instance, auxin-regulated gene involved in organ size and *PP2C* (Table 4). The total regulatory mechanism of NAA on the tuberous root yield and quality of *Rehmannia glutinosa* was illustrated in Figure 8.

In addition, catalpol as one of *R. glutinosa* quality markers was not annotated to a whole KEGG pathway in that there is not its complete biosynthesis pathway in the KEGG database until now. However, its biosynthesis pathway could be found in the terpenoid synthesis pathway in *R. glutinosa*, which was divided into MVA pathway (mevalonate pathway) and MEP pathway (2-C-methyl-D-erythritol-4-phosphate) [17,59]. According to those key enzyme genes in its biosynthesis pathway, newly reported in *R. glutinosa* [17], we screened 11 DEG-encoding enzymes, taking part in the catalpol synthesis pathway, from 1113 DEGs, of which 9 genes were down-regulated and 2 genes were up-regulated. This result showed that the up-regulation of catalpol content, detected by HPLC, may be due to both up-regulated cytochrome P450 reductase genes or those up- and down-regulated DEGs working together.

## 5. Conclusions

To sum up, exogenous NAA could increase the tuberous root yield of *R. glutinosa* in its field production by modifying the yield related indices of its tuberous roots and change the accumulation of its metabolites influencing its quality. Its regulatory mechanism on the tuberous root yield and quality was that the up-regulation or down-regulation of ARFs by NAA up-regulated or down-regulated DEGs. This study provided some valuable resources, such as tuberous root indices, key genes, and DAMs of *R. glutinosa* in response to NAA for distinguishing the CGs from NTs, and novel insights into the regulatory mechanism of NAA effects on both at the transcriptomic and metabolomic levels, so it will lay a theoretical foundation for NAA-regulated plant yield and quality, and provide references for prohibiting the uses of NAA as a swelling agents in medicinal tuber plants in China [60]. However, the regulatory mechanism of NAA effects on its yield related indices and metabolites will be discussed in more detail in the future.

## Figures and Tables

**Table 1:** The comparison between the morphological indices of NT and CG.

Sample	Root Mass Per Plant/g	Root Number Per Plant	The Longest Root Length/cm	The Thickest Root Diameter/cm	Mean Root Diameter Per Plant/cm
CK	212.02 ± 70.35 ^a^	6.00 ± 1.21 ^b^	18.87 ± 4.57 ^c^	3.40 ± 0.71 ^d^	2.32 ± 0.17 ^e^
NT	256.84 ± 78.66 ^a′^	12.00 ± 2.08 ^b′^	17.58 ± 0.24 ^c′^	3.20 ± 0.24 ^d′^	2.52 ± 0.34 ^e′^

^a^ and ^a^^′^ as well as ^b^ and ^b^^′^ (*p* < 0.01); ^c^ and ^c^^′^ (*p* < 0.05); ^d^ and ^d^^′^ as well as ^e^ and ^e^^′^ (*p* > 0.05).

**Table 2:** Determination of quality marker contents by HPLC.

Sample	Catalpol/%	Rehmannioside D/%	Acteoside/%
CK	0.7633 ± 0.0002 ^a^	0.2067 ± 0.0000 ^b^	0.0620 ± 0.0006 ^c^
NT	1.9867 ± 0.0003 ^a′^	0.2436 ± 0.0000 ^b′^	0.0717 ± 0.0000 ^c′^

^a^ and ^a^^′^ (*p* < 0.05); ^b^ and ^b^^′^ as well as ^c^ and ^c^^′^ (*p* > 0.05).

**Table 3:** Regulations of DEG(TF)s to DAMs via 10 common metabolic pathways.

TF Name	R_1_	Enzyme Name	R_2_	Pathway	DAM	R_3_
EIN3	up	ASP5, LIP1, CMT2, CYSD2	up	d, h, j	①	up
EIN3	up	PED, ACX1, CYP75B2, CCoAOMT, CHI,RG, RFS *, RFS6 *, SHM2, CS, ACO, MET	down	a, b, c, d, e, f, g, i	②	down
		agl, CMT2, CYSD2	up			
EIN3	up	CYP75B2, TAT, RFS *, LPD1, GS1-1, SQE1	down	b, d, e, f, g, h, j	③	up
		ASP5, CMT2, CYSD2	up			
EIN3	up	ACO	down	h	④	down
EIN3	down	PED, ACX1, CYP75B2, CcoAOMT, 4CL, TAT, unknow, RG, BGLU12 *, PER21, GOLS1, RFS *, RFS6 *, SHM2, CS, GS1-1, HD, PGDH2, SQE1, AOC3, SAMDC *, MET	down	a, b, c, d, e, f, g, h, i, j	⑤	up
		BGLU1 *	up			
EIN3	down	PED, ACX1, CYP75B2, CCoAOMT *, CHI, TAT, RG, BGLU12 *, RFS *, RFS6 *, SHM2, CS, ACO, HD, PGDH2, MET	down	a, b, c, d, e, f, g, i, j	⑥	down
		ASP5, CMT2, CYSD2	up			
EIN3	down	PED, ACX1, CYP75B2, CcoAOMT *, CHI, 4CL *, unknow, RG, BGLU12 *, PER21, GOLS1, RFS *, RFS6 *, SHM2, CS, ACO, HD, PGDH2, SQE1, AOC3, SAMDC *, MET	down	a, b, c, d, e, f, g, h, i, j	⑦	down
		CYSD2	up			
BZR1	up	ASP5, LIP1, CMT2, CYSD2	up	d, h, j	①	up
BZR1	up	ACO	down	h	④	down
BZR1	up	PED, ACX1, CYP75B2, CCoAOMT *, CHI, RG, RFS *, RFS6 *, SHM2, CS, ACO, MET	down	a, b, c, d, e, f, g, i	②	down
		agl, CMT2, CYSD2	up			
BZR1	up	CYP75B2, TAT, RFS *, LPD1, GS1-1, SQE1	down	b, d, e, f, g, h, j	③	up
		ASP5, CMT2, CYSD2	up			
Aux/IAA	up	ASP5, LIP1, CMT2, CYSD2	up	d, h, j	①	up
Aux/IAA	up	CYP75B2, TAT, RFS *, LPD1, GS1-1, SQE1	down	b, d, e, f, g, h, j	③	up
		ASP5, CMT2, CYSD2	up			
		ASP5, CMT2, CYSD2	up			

① beta-sitosterol. ② S-adenosylmethionine. ③ 3-hydroxybenzoic acid. ④ Naringin. ⑤ Glyoxylic acid. ⑥ Sinapoyl aldehyde. ⑦ 5,7-dihydroxyflavone. a. Biosynthesis of unsaturated fatty acids; b. Flavone and flavonol biosynthesis; c. Phenylpropanoid biosynthesis; d. Cysteine and methionine metabolism; e. Galactose metabolism; f. Glyoxylate and dicarboxylate metabolism; g. Glycine, serine and threonine metabolism; h. Steroid biosynthesis; i. alpha-Linolenic acid metabolism; j. arginine and proline metabolism. EIN3: ethylene-insensitive protein 3; BZR1: Brassinosteroid resistant 1/2; Aux/IAA protein: auxin/indole-3-acetic acid protein. ASP5: Aspartate aminotransferase; LIP1: Triacylglycerol lipase 1; CMT2: DNA (cytosine-5)-methyltransferase CMT2; CYSD2: Bifunctional L-3-cyanoalanine synthase/cysteine synthase D2; PED: 3-ketoacyl-CoA thiolase 2; ACX1: Peroxisomal acyl-coenzyme A oxidase 1; CYP75B2: Flavonoid 3′-monooxygenase; CCoAOMT: caffeoyl-CoA O-methyltransferase; CHI: Chalcone-flavononeisomerase; RG: Raucaffricine-O-beta-D-glucosidase; RFS: Galactinol-sucrose galactosyltransferase; agl: Alpha-galactosidase; RFS6: galactinol-sucrose galactosyltransferase 6; SHM2: Serine hydroxymethyltransferase 2; CS: Citrate synthase; ACO: Aconitatehydratase; MET: 5-methyltetrahydropteroyltriglutamate-homocysteinemethyltransferase; TAT: aminotransferase TAT2; LPD1: Dihydrolipoyl dehydrogenase 1; GS1-1: Glutamine synthetase cytosolic isozyme 1; SQE1: Squalene monooxygenase; 4CL: 4-coumarate-CoA ligase 2; BGLU1: Beta-glucosidase 1; BGLU12: Beta-glucosidase 12; PER21: Peroxidase 21; GOLS1: Galactinol synthase 1; HD: Homoserine dehydrogenase; PGDH2: D-3-phosphoglycerate dehydrogenase 2; AOC3: Allene oxide cyclase 3; SAMDC: S-adenosylmethionine decarboxylase beta chain. *: Genes related to both quality and yield. R_1_: TF regulation. R_2_: DEG regulation. R_3_: DAM regulation. EIN3genes: F01_transcript_42053, F01_transcript_9341; F01_transcript_42053; F01_transcript_88073. BZR1genes: F01_transcript_54031, F01_transcript_17041. Aux/IAA genes: F01_transcript_21517.

**Table 4:** Regulation of DEGs associated with yield on yield-related factors.

Gene Description	Gene ID	Yield Related Factors	References
beta-glucosidase	F01_transcript_45042, F01_transcript_14367, F01_transcript_57187, F01_transcript_57503, F01_transcript_79209, F01_transcript_83451, F01_transcript_86382, F01_transcript_88550, F01_transcript_45952, F01_transcript_48883	biomass	[18]
4-coumarate--CoA ligase	F01_transcript_33286	length	[20]
caffeoyl-CoA O-methyltransferase	F01_transcript_54233, F01_transcript_88383	biomass	[19]
protein phosphatase 2C	F01_transcript_10164, F01_transcript_60137, F01_transcript_40403	branching root numbers	[36]
brassinosteroid resistant 1/2	F01_transcript_54031, F01_transcript_17041, F01_transcript_16804	branching root numbers	[37]
auxin responsive GH3 gene family	F01_transcript_99345	branching root numbers	[41]
Auxin-regulated gene involved in organ size	F01_transcript_35805, F01_transcript_46007	width (thickening)	[39]
Expansin-like A1	F01_transcript_22738	width (thickening)	[42]
Lipoxygenase	F01_transcript_63376, F01_transcript_80652	width (thickening)	[43]
MADS-box	F01_transcript_48636	width (thickening)	[44]
Allantoinase	F01_transcript_32117, F01_transcript_15391, F01_transcript_66084, F01_transcript_14633, F01_transcript_48348, F01_transcript_98250	branching root numbers	[38]
Probable starch synthase 4	F01_transcript_2028	biomass	[35]
Galactinol-sucrose galactosyltransferase	F01_transcript_5582, F01_transcript_97134, F01_transcript_6840	biomass	[35]
Auxin response factor	F01_transcript_46036, F01_transcript_79445	length	[40]

**Table 5:** TF genes for auxin signal transduction pathway and their expression regulation.

ID	Gene	Regulation	CG-Mean (FPKM)	NT-Mean (FPKM)
F01_transcript_4608	TIR1	normal	9.54	9.85
F01_transcript_5273		normal	0.13	0.18
F01_transcript_5421		normal	10.59	9.12
F01_transcript_83028		normal	7.32	6.19
F01_transcript_86411		normal	0.42	0.46
F01_transcript_89970		normal	12.59	10.31
F01_transcript_73082		normal	7.56	6.82
F01_transcript_18581	SCF	up	14.96	23.54
F01_transcript_19200		up	7.27	19.75
F01_transcript_23979		up	86.73	176.44
F01_transcript_41747		up	17.40	29.12
F01_transcript_47554		up	2.86	5.73
F01_transcript_49230		up	12.32	26.83
F01_transcript_4394		up	10.93	17.55
F01_transcript_4877		up	9.03	15.48
F01_transcript_82073		up	1.95	4.66
F01_transcript_83142		up	2.59	5.14
F01_transcript_83618		up	0.79	2.11
F01_transcript_91955		up	12.04	18.84
F01_transcript_79445	ARF1	up	1.41	3.73
F01_transcript_46036	ARF2	down	7.73	2.64
F01_transcript_21517	AUX/IAA	up	14.40	26.55

## Data Availability

The raw data of all RNA-Seq samples obtained in this study were deposited in the NCBI Sequence Read Archive under the project with identification number PRJNA780233. The metabolomics data that support the findings of this study have been deposited into CNGB Sequence Archive (CNSA) of China National GeneBank DataBase (CNGBdb) with accession number CNP0003233.

## Supplementary Materials

The following supporting information can be downloaded at: https://www.mdpi.com/article/10.3390/cimb44080227/s1, Figure S1: Metabolomics analysis of *R. glutinosa* NTs and CGs. (a) PCA score plots for all samples. (b) PLS-DA score plots for all samples. (c) OPLS-DA score plots for all samples. (d) Heatmap clustering of 29 DAMs. There are six biological repeats in each group; Figure S2: Statistical histogram of DAMs identified by primary mass spectrometry; Figure S3: Histogram of several DAMs identified by secondary mass spectrometry; Figure S4: The number of the metabolites in every class; Figure S5: DEGs volcano plot; Figure S6: GO annotation analysis of DEGs; Figure S7: KEGG annotation analysis of DEGs based on enlarged lowest Q-value; Table S1: NAA-treated *R. glutinosa* and untreated yield; Table S2: List of 29 DAMs identified by secondary mass spectrometry; Table S3: Evaluation statistics list of sample sequencing clean data; Table S4: Annotation information of DEGs; Table S5: Correlation and regulation of DEGs to DAMs via KEGG pathways; Table S6: Correlation and regulation of DEGs belonging to transcription factors (TFs) to DAMs; Table S7: List of RT-qPCR primers.
