# Supplementary material for: The Effects of NAA on the Tuberous Root Yield and Quality of Rehmannia glutinosa and Its Regulatory Mechanism by Transcriptome and Metabolome Profiling
Source: Curr Issues Mol Biol. 2022 Jul 22;44(8):3291–311. doi: 10.3390/cimb44080227 (PMC9394425; doi:10.3390/cimb44080227)
Supplement: Supplementary file 1 [file cimb-44-00227-s001.zip › cimb-1803922-supplementary.pdf]

Figure S1. Metabolomics analysis of *R. glutinosa* NTs and CGs. (a) PCA score plots for all samples. (b)PLS-DA score plots for all samples. (c)OPLS-DA score plots for all samples. (d)Heatmap clustering of 29 DAMs. There are six biological repeats in each group.

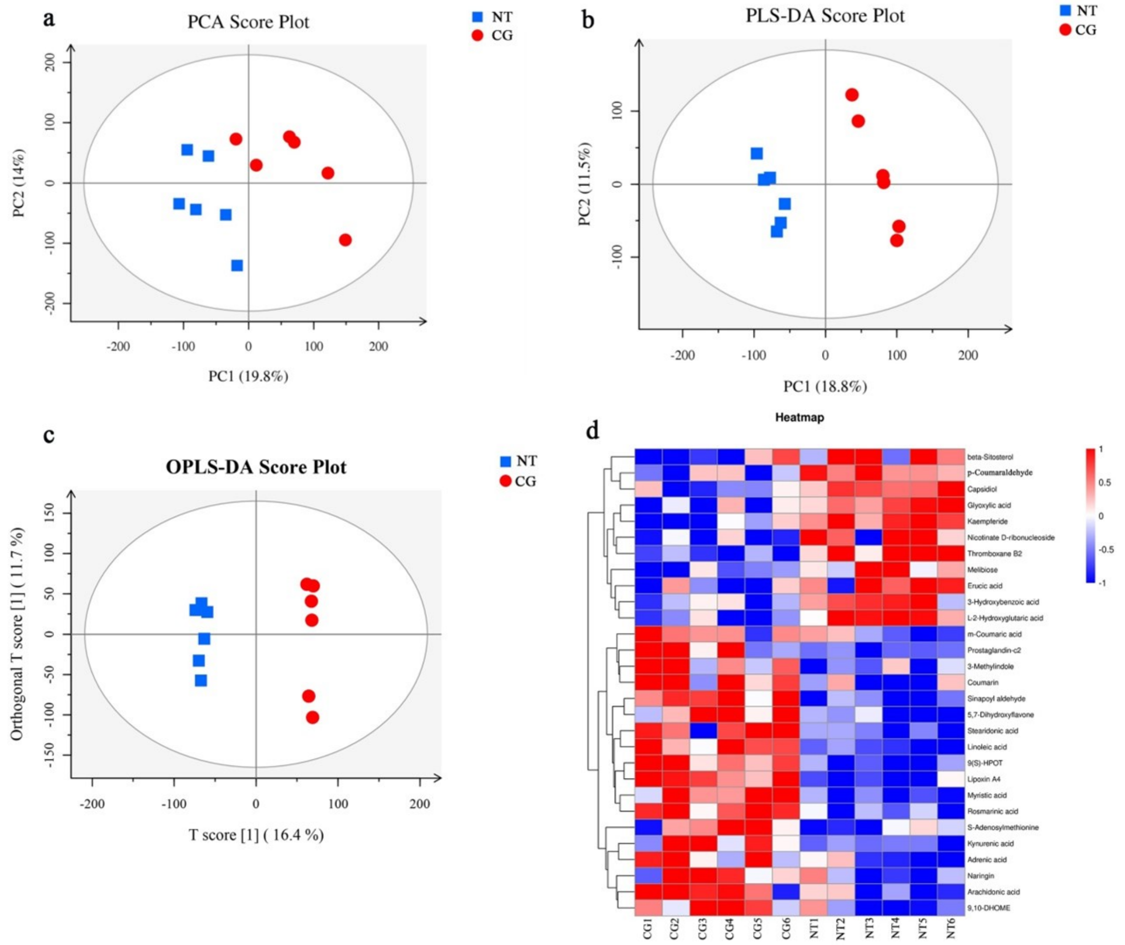

Figure S2 Statistical histogram of DAMs identified by primary mass spectrometry

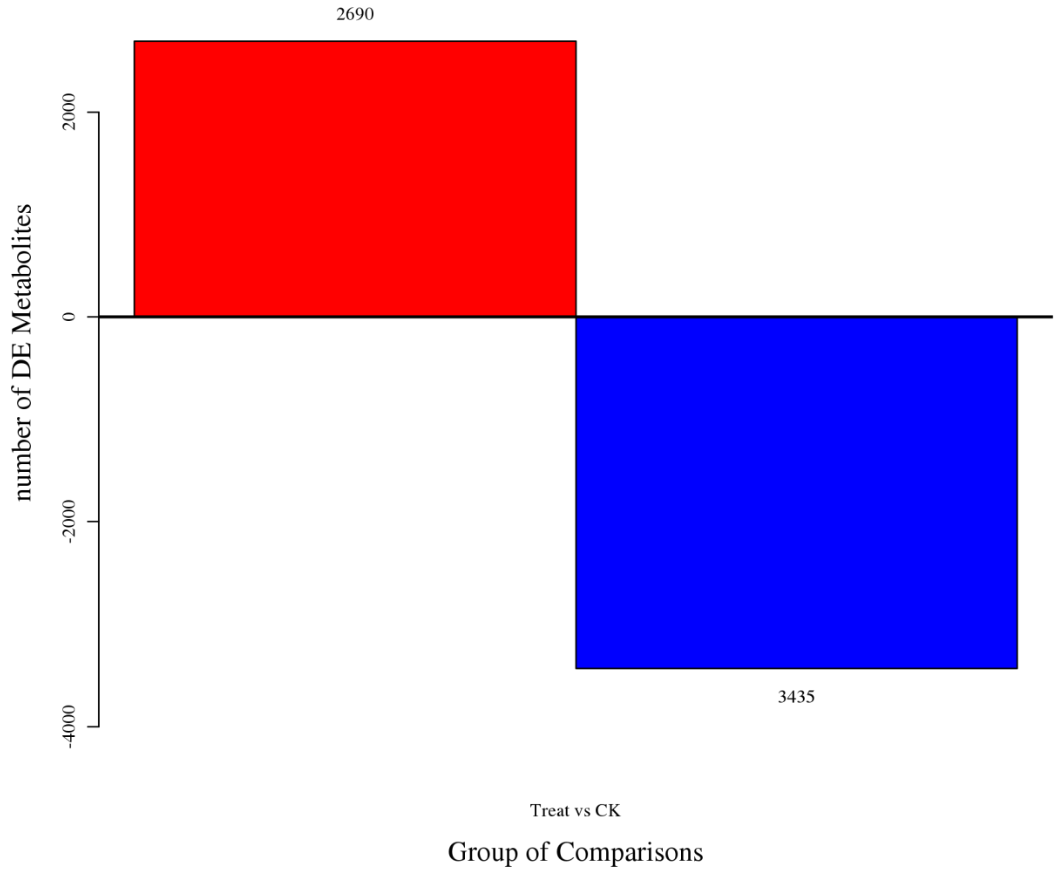

Figure S3. Histogram of several DAMs identified by secondary mass spectrometry

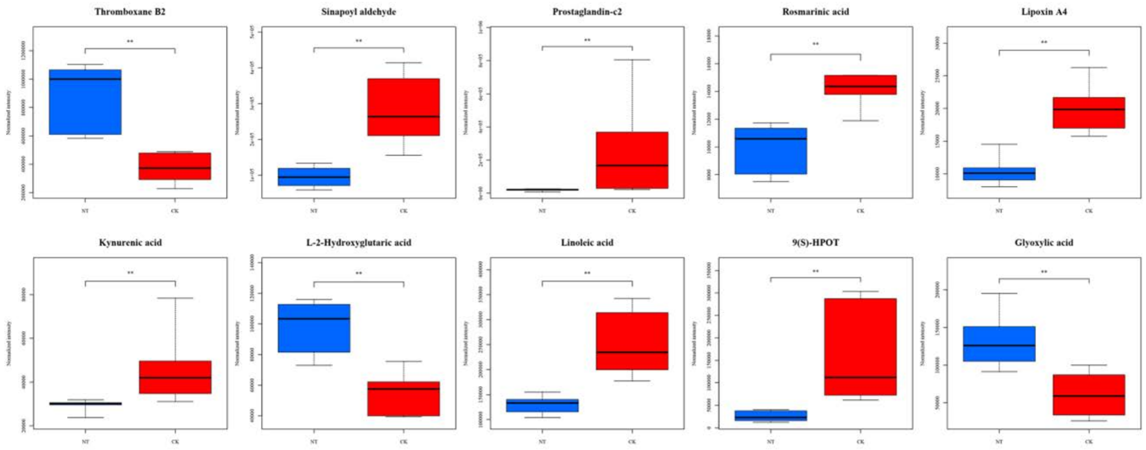

Figure S4. The number of the metabolites in every class

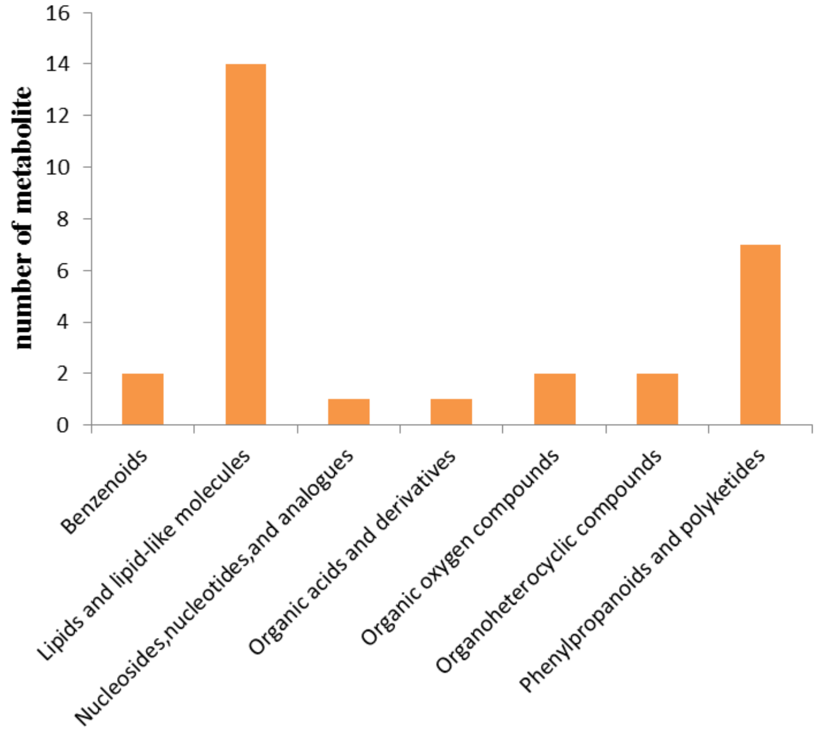

Figure S5. DEGs volcano plot

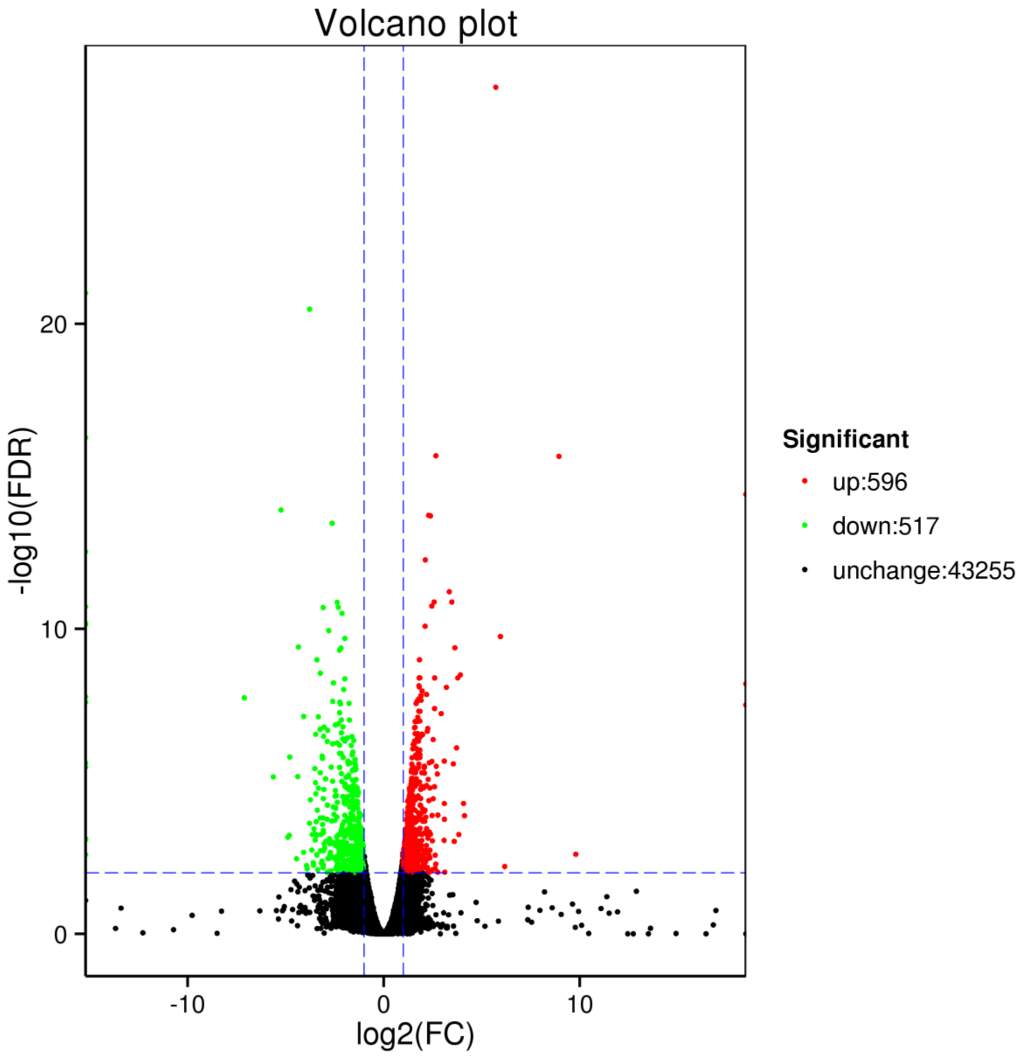

Figure S6. GO annotation analysis of DEGs

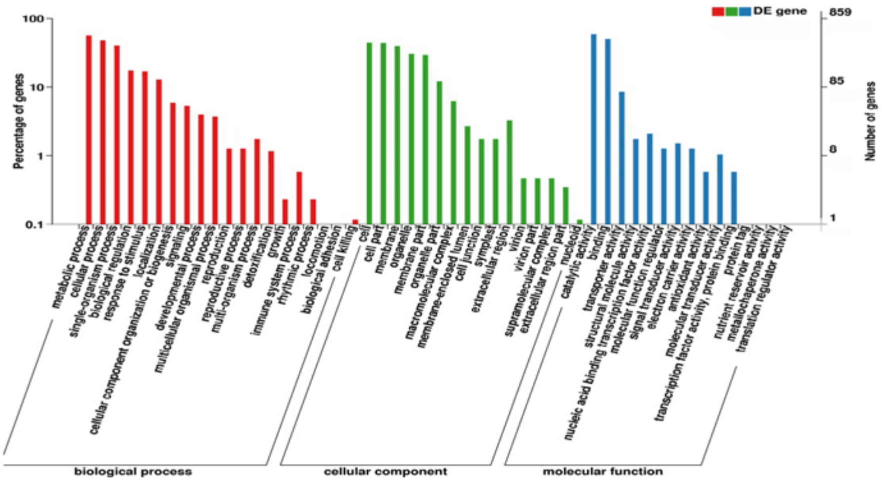

Figure S7. KEGG annotation analysis of DEGs based on enlarged lowest Q-value

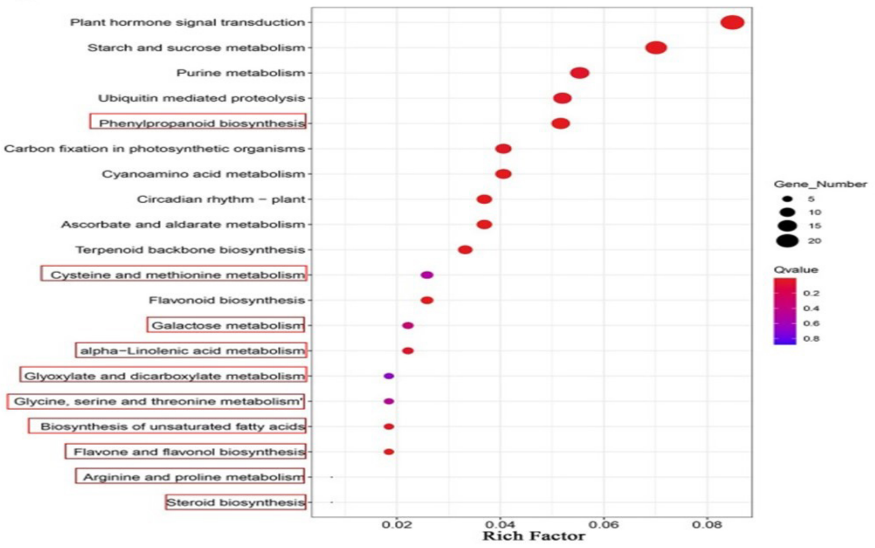

Table S1. NAA-treated *R. glutinosa* and untreated yield.

sample	Yield of 1 mu (kg)	Equivalent yield (kg/hm^2)	Yield increase/%
CK	2345.17	35017.50	
NT	2651.17	39569.78	13

TableS2. List of 29 DAMs identified by secondary mass spectrometry

name	mz	rt	ppm	KEGG	FC	log2(FC)	p.value	FDR	regu-lartion	pos-neg
p-Coumaraldehyde	149.06	656.69	1.87	C05608	1.874	0.906	0.005	0.178	up	pos
3-Hydroxybenzoic acid	137.06	106.25	14.26	C00587	2.456	1.296	0.020	0.223	up	neg
3-Methylindole	132.08	284.58	6.94	C08313	0.308	-1.698	0.031	0.231	down	pos
5,7-Dihydroxyflavone	255.06	545.86	3.78	C10028	0.378	-1.403	0.008	0.178	down	pos
9(S)-HPOT	293.21	806.17	1.17	C16321	0.158	-2.659	0.005	0.178	down	pos
9,10-DHOME	313.24	465.77	5.29	C14828	0.392	-1.350	0.013	0.202	down	neg
Adrenic acid	331.26	558.61	14.15	C16527	0.649	-0.623	0.045	0.286	down	neg
Arachidonic acid	303.24	901.98	14.73	C00219	0.078	-3.689	0.031	0.248	down	neg
beta-Sitosterol	397.39	668.82	6.86	C01753	1.977	0.983	0.045	0.264	up	pos
Capsidiol	219.17	980.66	0.01	C09627	1.612	0.689	0.008	0.178	up	pos
Coumarin	147.04	369.17	3.58	C05851	0.474	-1.077	0.045	0.264	down	pos
Erucic acid	338.34	1059.15	1.55	C08316	1.760	0.816	0.020	0.206	up	pos
Glyoxylic acid	74.10	323.07	11.27	C00048	2.172	1.119	0.008	0.178	up	pos
Kaempferide	301.08	681.16	11.26	C10098	1.610	0.687	0.005	0.178	up	pos
Kynurenic acid	190.09	435.02	2.74	C01717	0.635	-0.656	0.008	0.178	down	pos
L-2-Hydroxyglutaric acid	149.04	901.79	3.57	C03196	1.774	0.827	0.008	0.178	up	pos
Linoleic acid	281.25	805.04	2.81	C01595	0.520	-0.943	0.005	0.178	down	pos
Lipoxin A4	351.22	822.79	5.96	C06314	0.521	-0.940	0.005	0.202	down	neg
m-Coumaric acid	165.05	484.09	0.86	C12621	0.568	-0.816	0.045	0.264	down	pos

Melibiose	343.14	93.76	8.00	C05400	2.048	1.034	0.013	0.184	up	pos
Myristic acid	228.21	557.86	3.29	C06424	0.206	-2.278	0.005	0.178	down	pos
Naringin	581.18	438.18	9.58	C09789	0.425	-1.235	0.045	0.264	down	pos
Nicotinate D-ribonucleoside	256.08	87.25	0.36	C05841	2.019	1.013	0.045	0.264	up	pos
Prostaglandin-c2	335.22	775.12	0.32	C05955	0.075	-3.736	0.008	0.178	down	pos
Rosmarinic acid	360.13	149.73	7.27	C01850	0.706	-0.502	0.005	0.202	down	neg
S-Adenosylmethionine	398.13	486.86	10.53	C00019	0.497	-1.010	0.045	0.264	down	pos
Sinapoyl aldehyde	209.08	377.34	0.04	C05610	0.342	-1.549	0.005	0.178	down	pos
Stearidonic acid	277.22	841.89	3.32	C16300	0.576	-0.797	0.031	0.231	down	pos
Thromboxane B2	353.23	557.24	0.61	C05963	2.401	1.264	0.005	0.178	up	pos

Table S3. Evaluation statistics list of sample sequencing clean data

Sample	Read Number	Base Number(b)	GC Content	≥Q30(%)
CK1	25,394,501	7,586,653,728	44.95	94.45
CK2	24,660,897	7,367,697,804	44.62	94.37
CK3	22,069,180	6,595,223,742	45.03	93.99
NT1	20,878,753	6,246,639,586	44.15	94.78
NT2	24,130,381	7,203,395,870	44.60	93.96
NT3	26,463,449	7,906,338,666	44.41	94.26
Total		42.91Gb		

Read Number：Total number of pair-end reads among clean data. Base Number：Total base number of clean Data. GC Content：G+C% Base Number. ≥Q30(%)：The percentage of mass value of clean data is greater than or equal to 30.

Table S4. Annotation information of DEGs

Databases	COG	GO	KEGG	KOG	Pfam	Swiss-Prot	NOG	NR	Total
Gene	534	859	454	693	926	867	1038	1076	1113
proportion/%	47.98	77.18	40.79	62.26	83.20	77.90	93.26	96.68	100

TableS5 Correlation and regulation of DEGs to DAMs via KEGG pathways

TableS5.1 Correlation and regulation of DEGs to DAMs via biosynthesis of unsaturated fatty acids

Gene ID	Regulation	Metabolite	Regulation
F01_transcript_16402	down	Glyoxylic acid	up
F01_transcript_16402	down	5,7-Dihydroxyflavone	down
F01_transcript_52404	down	Glyoxylic acid	up
F01_transcript_52404	down	S-Adenosylmethionine	down
F01_transcript_52404	down	Sinapoyl aldehyde	down
F01_transcript_52404	down	5,7-Dihydroxyflavone	down
F01_transcript_64411	down	Glyoxylic acid	up
F01_transcript_64411	down	S-Adenosylmethionine	down
F01_transcript_64411	down	Sinapoyl aldehyde	down
F01_transcript_6441	down	5,7-Dihydroxyflavone	down

1			
F01_transcript_8765	down	Glyoxylic acid	up
F01_transcript_8765	down	Sinapoyl aldehyde	down
F01_transcript_8765	down	5,7-Dihydroxyflavone	down
F01_transcript_98354	down	Glyoxylic acid	up
F01_transcript_98354	down	Sinapoyl aldehyde	down
F01_transcript_98354	down	5,7-Dihydroxyflavone	down

F01_transcript_16402 : 3-ketoacyl-CoA thiolase 2, peroxisomal; F01_transcript_52404: 3-ketoacyl-CoA thiolase 2; F01_transcript_64411: Peroxisomal acyl-coenzyme A oxidase 1; F01_transcript_8765: Peroxisomal acyl-coenzyme A oxidase 1; F01_transcript_98354: 3-ketoacyl-CoA thiolase 2

TableS5.2 Correlation and regulation of DEGs to DAMs via flavone and flavonol biosynthesis

Gene ID	Regulation	Metabolite	Regulation
F01_transcript_13718	down	S-Adenosylmethionine	down
F01_transcript_13718	down	Sinapoyl aldehyde	down
F01_transcript_13718	down	5,7-Dihydroxyflavone	down
F01_transcript_13718	down	Kynurenic acid	down
F01_transcript_36219	down	Myristic acid	down
F01_transcript_36219	down	S-Adenosylmethionine	down
F01_transcript_36219	down	Sinapoyl aldehyde	down
F01_transcript_36219	down	5,7-Dihydroxyflavone	down
F01_transcript_36219	down	Kynurenic acid	down
F01_transcript_65146	down	Glyoxylic acid	up
F01_transcript_65146	down	S-Adenosylmethionine	down
F01_transcript_65146	down	Sinapoyl aldehyde	down
F01_transcript_65146	down	5,7-Dihydroxyflavone	down
F01_transcript_76506	down	Glyoxylic acid	up
F01_transcript_76506	down	S-Adenosylmethionine	down
F01_transcript_76506	down	Sinapoyl aldehyde	down
F01_transcript_76506	down	5,7-Dihydroxyflavone	down
F01_transcript_76506	down	3-Hydroxybenzoic acid	up
F01_transcript_93751	up	9,10-DHOME	down

F01_transcript_13718: Flavonoid 3'-monooxygenase; F01_transcript_36219: Flavonoid 3'-monooxygenase; F01_transcript_65146: Flavonoid 3'-monooxygenase; F01_transcript_76506: Flavonoid 3'-monooxygenase; F01_transcript_93751: Flavonoid 3',5'-methyltransferase

TableS5.3 Correlation and regulation of DEGs to DAMs via flavonoid biosynthesis

Gene ID	Regulation	Metabolite	Regulation
F01_transcript_13718	down	S-Adenosylmethionine	down
F01_transcript_13718	down	Sinapoyl aldehyde	down
F01_transcript_13718	down	5,7-Dihydroxyflavone	down
F01_transcript_13718	down	Kynurenic acid	down
F01_transcript_36219	down	Myristic acid	down
F01_transcript_36219	down	S-Adenosylmethionine	down
F01_transcript_36219	down	Sinapoyl aldehyde	down
F01_transcript_36219	down	5,7-Dihydroxyflavone	down
F01_transcript_36219	down	Kynurenic acid	down
F01_transcript_54233	down	Glyoxylic acid	up
F01_transcript_54233	down	S-Adenosylmethionine	down
F01_transcript_54233	down	Sinapoyl aldehyde	down
F01_transcript_54233	down	5,7-Dihydroxyflavone	down
F01_transcript_65068	down	S-Adenosylmethionine	down
F01_transcript_65068	down	Sinapoyl aldehyde	down
F01_transcript_65068	down	5,7-Dihydroxyflavone	down
F01_transcript_65146	down	Glyoxylic acid	up
F01_transcript_65146	down	S-Adenosylmethionine	down
F01_transcript_65146	down	Sinapoyl aldehyde	down
F01_transcript_65146	down	5,7-Dihydroxyflavone	down
F01_transcript_76506	down	Glyoxylic acid	up
F01_transcript_76506	down	S-Adenosylmethionine	down
F01_transcript_76506	down	Sinapoyl aldehyde	down
F01_transcript_76506	down	5,7-Dihydroxyflavone	down
F01_transcript_7650	down	3-Hydroxybenzoic acid	up

6			
F01_transcript_88383	down	Glyoxylic acid	up
F01_transcript_88383	down	S-Adenosylmethionine	down
F01_transcript_88383	down	Sinapoyl aldehyde	down
F01_transcript_88383	down	5,7-Dihydroxyflavone	down

F01_transcript_13718: Flavonoid 3'-monooxygenase; F01_transcript_36219: Flavonoid 3'-monooxygenase; F01_transcript_54233: Caffeoyl-CoA O-methyltransferase; F01_transcript_65068: Chalcone--flavonone isomerase; F01_transcript_65146: Flavonoid 3'-monooxygenase; F01_transcript_76506: Flavonoid 3'-monooxygenase; F01_transcript_88383: Caffeoyl-CoA O-methyltransferase

TableS5.4 Correlation and regulation of DEGs to DAMs via phenylalanine metabolism

Gene ID	Regulation	Metabolite	Regulation
F01_transcript_33286	down	Glyoxylic acid	up
F01_transcript_33286	down	5,7-Dihydroxyflavone	down
F01_transcript_88383	down	Glyoxylic acid	up
F01_transcript_88383	down	S-Adenosylmethionine	down
F01_transcript_88383	down	Sinapoyl aldehyde	down
F01_transcript_88383	down	5,7-Dihydroxyflavone	down
F01_transcript_50638	down	Glyoxylic acid	up
F01_transcript_50638	down	9(S)-HPOT	down
F01_transcript_50638	down	Kaempferide	up
F01_transcript_50638	down	3-Methylindole	down
F01_transcript_50638	down	(E)-3-(4-Hydroxyphenyl)-2-propenal	up
F01_transcript_50638	down	Sinapoyl aldehyde	down
F01_transcript_50638	down	3-Hydroxybenzoic acid	up
F01_transcript_13137	down	Glyoxylic acid	up
F01_transcript_13137	down	5,7-Dihydroxyflavone	down
F01_transcript_49410	up	beta-Sitosterol	up
F01_transcript_49410	up	(E)-3-(4-Hydroxyphenyl)-2-propenal	up
F01_transcript_49410	up	Sinapoyl aldehyde	down
F01_transcript_49410	up	3-Hydroxybenzoic acid	up
F01_transcript_54233	down	Glyoxylic acid	up
F01_transcript_54233	down	S-Adenosylmethionine	down
F01_transcript_54233	down	Sinapoyl aldehyde	down
F01_transcript_54233	down	5,7-Dihydroxyflavone	down

F01_transcript_33286: 4-coumarate--CoA ligase 2; F01_transcript_88383: Caffeoyl-CoA O-methyltransferase ; F01_transcript_50638: aminotransferase TAT2; F01_transcript_13137: Primary amine oxidase; F01_transcript_49410: Aspartate aminotransferase; F01_transcript_54233: Caffeoyl-CoA O-methyltransferase

TableS5.5 Correlation and regulation of DEGs to DAMs via phenylpropanoid biosynthesis

Gene ID	Regulation	Metabolite	Regulation
F01_transcript_45042	down	Glyoxylic acid	up
F01_transcript_45042	down	Sinapoyl aldehyde	down
F01_transcript_45042	down	5,7-Dihydroxyflavone	down
F01_transcript_45952	down	Glyoxylic acid	up
F01_transcript_45952	down	5,7-Dihydroxyflavone	down
F01_transcript_48883	down	Glyoxylic acid	up
F01_transcript_48883	down	S-Adenosylmethionine	down

F01_transcript_48883	down	Sinapoyl aldehyde	down
F01_transcript_48883	down	5,7-Dihydroxyflavone	down
F01_transcript_57503	down	Glyoxylic acid	up
F01_transcript_57503	down	Sinapoyl aldehyde	down
F01_transcript_57503	down	5,7-Dihydroxyflavone	down
F01_transcript_79209	down	Glyoxylic acid	up
F01_transcript_79209	down	Sinapoyl aldehyde	down
F01_transcript_79209	down	5,7-Dihydroxyflavone	down
F01_transcript_83451	down	Glyoxylic acid	up
F01_transcript_83451	down	Sinapoyl aldehyde	down
F01_transcript_83451	down	5,7-Dihydroxyflavone	down
F01_transcript_86382	down	Glyoxylic acid	up
F01_transcript_86382	down	5,7-Dihydroxyflavone	down
F01_transcript_88550	down	Glyoxylic acid	up
F01_transcript_88550	down	5,7-Dihydroxyflavone	down
F01_transcript_54233	down	Glyoxylic acid	up
F01_transcript_54233	down	S-Adenosylmethionine	down
F01_transcript_54233	down	Sinapoyl aldehyde	down
F01_transcript_54233	down	5,7-Dihydroxyflavone	down
F01_transcript_88383	down	Glyoxylic acid	up
F01_transcript_88383	down	S-Adenosylmethionine	down
F01_transcript_88383	down	Sinapoyl aldehyde	down
F01_transcript_88383	down	5,7-Dihydroxyflavone	down
F01_transcript_14367	up	Glyoxylic acid	up
F01_transcript_14367	up	Kaempferide	up
F01_transcript_14367	up	Lipoxin A4	down
F01_transcript_14367	up	Rosmarinic acid	down
F01_transcript_57187	down	Glyoxylic acid	up
F01_transcript_57187	down	Sinapoyl aldehyde	down
F01_transcript_57187	down	5,7-Dihydroxyflavone	down
F01_transcript_94508	down	Glyoxylic acid	up
F01_transcript_94508	down	5,7-Dihydroxyflavone	down
F01_transcript_33286	down	Glyoxylic acid	up
F01_transcript_33286	down	5,7-Dihydroxyflavone	down

F01_transcript_45042: Raucaffricine-O-beta-D-glucosidase; F01_transcript_45952: Raucaffricine-O-beta-D-glucosidase; F01_transcript_48883: Raucaffricine-O-beta-D-glucosidase; F01_transcript_57503: Raucaffricine-O-beta-D-glucosidase; F01_transcript_79209: Raucaffricine-O-beta-D-glucosidase; F01_transcript_83451: Raucaffricine-O-beta-D-glucosidase; F01_transcript_86382: Raucaffricine-O-beta-D-glucosidase; F01_transcript_88550: Raucaffricine-O-beta-D-glucosidase; F01_transcript_54233: Caffeoyl-CoA O-methyltransferase; F01_transcript_88383: Caffeoyl-CoA O-methyltransferase; F01_transcript_14367: Beta-glucosidase 1; F01_transcript_57187: Beta-glucosidase 12; F01_transcript_94508: Peroxidase 21; F01_transcript_33286: 4-coumarate--CoA ligase 2

TableS5.6 Correlation and regulation of DEGs to DAMs via Cysteine and methionine metabolism

Gene ID	Regulation	Metabolite	Regulation
F01_transcript_92037	up	beta-Sitosterol	up
F01_transcript_92037	up	S-Adenosylmethionine	down
F01_transcript_92037	up	Sinapoyl aldehyde	down
F01_transcript_92037	up	3-Hydroxybenzoic acid	up
F01_transcript_66132	up	beta-Sitosterol	up
F01_transcript_66132	up	S-Adenosylmethionine	down
F01_transcript_66132	up	Sinapoyl aldehyde	down
F01_transcript_66132	up	5,7-Dihydroxyflavone	down
F01_transcript_66132	up	3-Hydroxybenzoic acid	up

F01_transcript_49410	up	beta-Sitosterol	up
F01_transcript_49410	up	(E)-3-(4-Hydroxyphenyl)-2-propenal	up
F01_transcript_49410	up	Sinapoyl aldehyde	down
F01_transcript_49410	up	3-Hydroxybenzoic acid	up
F01_transcript_3160	down	Glyoxylic acid	up
F01_transcript_3160	down	Sinapoyl aldehyde	down
F01_transcript_3160	down	5,7-Dihydroxyflavone	down
F01_transcript_65625	down	Glyoxylic acid	up
F01_transcript_65625	down	S-Adenosylmethionine	down
F01_transcript_65625	down	Sinapoyl aldehyde	down
F01_transcript_65625	down	5,7-Dihydroxyflavone	down
F01_transcript_14387	down	Glyoxylic acid	up
F01_transcript_14387	down	5,7-Dihydroxyflavone	down
F01_transcript_50638	down	Glyoxylic acid	up
F01_transcript_50638	down	Kaempferide	up
F01_transcript_50638	down	3-Methylindole	down
F01_transcript_50638	down	9(S)-HPOT	down
F01_transcript_50638	down	(E)-3-(4-Hydroxyphenyl)-2-propenal	up
F01_transcript_50638	down	Sinapoyl aldehyde	down
F01_transcript_50638	down	3-Hydroxybenzoic acid	up

F01_transcript_92037: DNA (cytosine-5)-methyltransferase CMT2，F01_transcript_66132: Bifunctional L-3-cyanoalanine synthase/cysteine synthase D2，F01_transcript_49410: Aspartate aminotransferase，F01_transcript_3160: Homoserine dehydrogenase，F01_transcript_65625: 5-methyltetrahydropteroyltriglutamate--homocysteine methyltransferase，F01_transcript_14387: S-adenosylmethionine decarboxylase beta chain，F01_transcript_50638: aminotransferase TAT2.

TableS5.7 Correlation and regulation of DEGs to DAMs via Galactose metabolism

Gene ID	Regulation	Metabolite	Regulation
F01_transcript_16765	down	Glyoxylic acid	up
F01_transcript_16765	down	5,7-Dihydroxyflavone	down
F01_transcript_5582	down	Glyoxylic acid	up
F01_transcript_5582	down	Kaempferide	up
F01_transcript_5582	down	Prostaglandin-c2	down
F01_transcript_5582	down	3-Hydroxybenzoic acid	up
F01_transcript_6840	down	Glyoxylic acid	up
F01_transcript_6840	down	S-Adenosylmethionine	down
F01_transcript_6840	down	Sinapoyl aldehyde	down
F01_transcript_6840	down	5,7-Dihydroxyflavone	down
F01_transcript_97079	up	S-Adenosylmethionine	down
F01_transcript_97134	down	Glyoxylic acid	up
F01_transcript_97134	down	S-Adenosylmethionine	down
F01_transcript_97134	down	Sinapoyl aldehyde	down
F01_transcript_97134	down	5,7-Dihydroxyflavone	down

F01_transcript_16765:Galactinol synthase 1; F01_transcript_5582: Galactinol--sucrose galactosyltransferase; F01_transcript_6840: Galactinol--sucrose galactosyltransferase; F01_transcript_97079: Alpha-galactosidase; F01_transcript_97134: Galactinol--sucrose galactosyltransferase 6

TableS5.8 Correlation and regulation of DEGs to DAMs via Glyoxylate and dicarboxylate metabolism

Gene ID	Regulation	Metabolite	Regulation
F01_transcript_13584	down	Glyoxylic acid	up
F01_transcript_13584	down	S-Adenosylmethionine	down
F01_transcript_13584	down	Sinapoyl aldehyde	down
F01_transcript_13584	down	5,7-Dihydroxyflavone	down

F01_transcript_13967	down	Myristic acid	down
F01_transcript_13967	down	9(S)-HPOT	down
F01_transcript_13967	down	3-Methylindole	down
F01_transcript_13967	down	(E)-3-(4-Hydroxyphenyl)-2-propenal	up
F01_transcript_13967	down	3-Hydroxybenzoic acid	up
F01_transcript_14891	down	Glyoxylic acid	up
F01_transcript_14891	down	S-Adenosylmethionine	down
F01_transcript_14891	down	Sinapoyl aldehyde	down
F01_transcript_14891	down	5,7-Dihydroxyflavone	down
F01_transcript_4862	down	Glyoxylic acid	up
F01_transcript_4862	down	3-Hydroxybenzoic acid	up
F01_transcript_57595	down	S-Adenosylmethionine	down
F01_transcript_57595	down	Sinapoyl aldehyde	down
F01_transcript_57595	down	5,7-Dihydroxyflavone	down
F01_transcript_57595	down	Naringin	down
F01_transcript_57595	down	Kynurenic acid	down

F01_transcript_13584: Serine hydroxymethyltransferase 2; F01_transcript_13967: Dihydrolipoyl dehydrogenase 1; F01_transcript_14891: Citrate synthase; F01_transcript_4862: Glutamine synthetase cytosolic isozyme 1; F01_transcript_57595: Aconitate hydratase

TableS5.9 Correlation and regulation of DEGs to DAMs via Glycine, serine and threonine metabolism

Gene ID	Regulation	Metabolite	Regulation
F01_transcript_13137	down	Glyoxylic acid	up
F01_transcript_13137	down	5,7-Dihydroxyflavone	down
F01_transcript_13584	down	Glyoxylic acid	up
F01_transcript_13584	down	S-Adenosylmethionine	down
F01_transcript_13584	down	Sinapoyl aldehyde	down
F01_transcript_13584	down	5,7-Dihydroxyflavone	down
F01_transcript_13967	down	Myristic acid	down
F01_transcript_13967	down	9(S)-HPOT	down
F01_transcript_13967	down	3-Methylindole	down
F01_transcript_13967	down	(E)-3-(4-Hydroxyphenyl)-2-propenal	up
F01_transcript_13967	down	3-Hydroxybenzoic acid	up
F01_transcript_3160	down	Glyoxylic acid	up
F01_transcript_3160	down	Sinapoyl aldehyde	down
F01_transcript_3160	down	5,7-Dihydroxyflavone	down
F01_transcript_77388	down	Glyoxylic acid	up
F01_transcript_77388	down	Sinapoyl aldehyde	down
F01_transcript_77388	down	5,7-Dihydroxyflavone	down

F01_transcript_13137: Primary amine oxidase; F01_transcript_13584: Serine hydroxymethyltransferase 2; F01_transcript_13967: Dihydrolipoyl dehydrogenase 1; F01_transcript_3160: Homoserine dehydrogenase; F01_transcript_77388: D-3-phosphoglycerate dehydrogenase 2

TableS5.10 Correlation and regulation of DEGs to DAMs via Steroid biosynthesis

Gene ID	Regulation	Metabolite	Regulation
F01_transcript_18023	up	beta-Sitosterol	up
F01_transcript_53548	down	Glyoxylic acid	up
F01_transcript_53548	down	5,7-Dihydroxyflavone	down
F01_transcript_53548	down	3-Hydroxybenzoic acid	up

F01_transcript_18023: Triacylglycerol lipase 1; F01_transcript_53548: Squalene monooxygenase

TableS5.11 Correlation and regulation of DEGs to DAMs via alpha-Linolenic acid metabolism

Gene ID	Regulation	Metabolite	Regulation
F01_transcript_16402	down	Glyoxylic acid	up
F01_transcript_16402	down	5,7-Dihydroxyflavone	down
F01_transcript_52404	down	Glyoxylic acid	up
F01_transcript_52404	down	S-Adenosylmethionine	down
F01_transcript_52404	down	Sinapoyl aldehyde	down
F01_transcript_52404	down	5,7-Dihydroxyflavone	down
F01_transcript_59138	down	Glyoxylic acid	up
F01_transcript_59138	down	5,7-Dihydroxyflavone	down
F01_transcript_64411	down	Glyoxylic acid	up
F01_transcript_64411	down	S-Adenosylmethionine	down
F01_transcript_64411	down	Sinapoyl aldehyde	down
F01_transcript_64411	down	5,7-Dihydroxyflavone	down
F01_transcript_8765	down	Glyoxylic acid	up
F01_transcript_8765	down	Sinapoyl aldehyde	down
F01_transcript_8765	down	5,7-Dihydroxyflavone	down
F01_transcript_98354	down	Glyoxylic acid	up
F01_transcript_98354	down	Sinapoyl aldehyde	down
F01_transcript_98354	down	5,7-Dihydroxyflavone	down

F01_transcript_16402: 3-ketoacyl-CoA thiolase 2; F01_transcript_52404: 3-ketoacyl-CoA thiolase 2; F01_transcript_59138: Allene oxide cyclase 3; F01_transcript_64411: Peroxisomal acyl-coenzyme A oxidase 1; F01_transcript_8765: Peroxisomal acyl-coenzyme A oxidase 1; F01_transcript_98354: 3-ketoacyl-CoA thiolase 2

TableS5.12 Correlation and regulation of DEGs to DAMs via Arginine and proline metabolism

Gene ID	Regulation	Metabolite	Regulation
F01_transcript_14387	down	Glyoxylic acid	up
F01_transcript_14387	down	5,7-Dihydroxyflavone	down
F01_transcript_49410	up	beta-Sitosterol	up
F01_transcript_49410	up	(E)-3-(4-Hydroxyphenyl)-2-propenal	up
F01_transcript_49410	up	Sinapoyl aldehyde	down
F01_transcript_49410	up	3-Hydroxybenzoic acid	up

F01_transcript_14387: S-adenosylmethionine decarboxylase beta chain; F01_transcript_49410: Aspartate aminotransferase

TableS6. Correlation and regulation of DEGs belonging to transcription factors(TFs) to DAMs

TF ID	Regulation	Metabolite	Regulation
F01_transcript_14491	up	S-Adenosylmethionine	down
F01_transcript_14491	up	Sinapoyl aldehyde	down
F01_transcript_14819	up	beta-Sitosterol	up
F01_transcript_14819	up	S-Adenosylmethionine	down
F01_transcript_14819	up	(E)-3-(4-Hydroxyphenyl)-2-propenal	up
F01_transcript_14819	up	Sinapoyl aldehyde	down
F01_transcript_14819	up	3-Hydroxybenzoic acid	up
F01_transcript_16804	up	beta-Sitosterol	up
F01_transcript_16804	up	S-Adenosylmethionine	down
F01_transcript_16804	up	3-Hydroxybenzoic acid	up
F01_transcript_17041	up	beta-Sitosterol	up
F01_transcript_17041	up	S-Adenosylmethionine	down
F01_transcript_17041	up	3-Hydroxybenzoic acid	up
F01_transcript_18056	down	Glyoxylic acid	up
F01_transcript_18056	down	S-Adenosylmethionine	down
F01_transcript_18056	down	Sinapoyl aldehyde	down
F01_transcript_18056	down	5,7-Dihydroxyflavone	down
F01_transcript_24248	down	S-Adenosylmethionine	down
F01_transcript_24248	down	Sinapoyl aldehyde	down
F01_transcript_24248	down	5,7-Dihydroxyflavone	down
F01_transcript_24692	down	Glyoxylic acid	up
F01_transcript_24692	down	S-Adenosylmethionine	down
F01_transcript_24692	down	Sinapoyl aldehyde	down
F01_transcript_24692	down	5,7-Dihydroxyflavone	down
F01_transcript_25025	down	Glyoxylic acid	up
F01_transcript_25025	down	Sinapoyl aldehyde	down
F01_transcript_25025	down	5,7-Dihydroxyflavone	down
F01_transcript_31742	up	beta-Sitosterol	up
F01_transcript_33244	up	Glyoxylic acid	up
F01_transcript_33244	up	9(S)-HPOT	down
F01_transcript_33244	up	Kaempferide	up
F01_transcript_33244	up	3-Methylindole	down
F01_transcript_33244	up	Stearidonic acid	down
F01_transcript_33244	up	Prostaglandin-c2	down
F01_transcript_33244	up	3-Hydroxybenzoic acid	up
F01_transcript_33244	up	m-Coumaric acid	down
F01_transcript_34832	down	Glyoxylic acid	up
F01_transcript_34832	down	5,7-Dihydroxyflavone	down
F01_transcript_34879	up	9(S)-HPOT	down
F01_transcript_34879	up	Kaempferide	up
F01_transcript_34879	up	3-Methylindole	down
F01_transcript_34879	up	beta-Sitosterol	up
F01_transcript_34879	up	(E)-3-(4-Hydroxyphenyl)-2-propenal	up
F01_transcript_34879	up	Rosmarinic acid	down
F01_transcript_34879	up	3-Hydroxybenzoic acid	up
F01_transcript_42053	up	beta-Sitosterol	up
F01_transcript_42053	up	S-Adenosylmethionine	down
F01_transcript_42053	up	Naringin	down
F01_transcript_43678	up	beta-Sitosterol	up
F01_transcript_43678	up	3-Hydroxybenzoic acid	up
F01_transcript_48038	down	Glyoxylic acid	up
F01_transcript_48038	down	Sinapoyl aldehyde	down
F01_transcript_48038	down	5,7-Dihydroxyflavone	down
F01_transcript_48038	down	3-Hydroxybenzoic acid	up
F01_transcript_48636	down	Glyoxylic acid	up

F01_transcript_48636	down	Kaempferide	up
F01_transcript_48636	down	3-Hydroxybenzoic acid	up
F01_transcript_50082	down	Glyoxylic acid	up
F01_transcript_50082	down	Sinapoyl aldehyde	down
F01_transcript_50082	down	5,7-Dihydroxyflavone	down
F01_transcript_54031	up	beta-Sitosterol	up
F01_transcript_54031	up	Naringin	down
F01_transcript_57597	down	Glyoxylic acid	up
F01_transcript_57597	down	3-Hydroxybenzoic acid	up
F01_transcript_58757	up	beta-Sitosterol	up
F01_transcript_58757	up	S-Adenosylmethionine	down
F01_transcript_58757	up	(E)-3-(4-Hydroxyphenyl)-2-propenal	up
F01_transcript_58757	up	Sinapoyl aldehyde	down
F01_transcript_65141	up	Kaempferide	up
F01_transcript_65141	up	beta-Sitosterol	up
F01_transcript_65141	up	Rosmarinic acid	down
F01_transcript_65141	up	3-Hydroxybenzoic acid	up
F01_transcript_67070	up	Glyoxylic acid	up
F01_transcript_67070	up	Sinapoyl aldehyde	down
F01_transcript_67070	up	5,7-Dihydroxyflavone	down
F01_transcript_68607	up	beta-Sitosterol	up
F01_transcript_70090	up	9,10-DHOME	down
F01_transcript_70090	up	beta-Sitosterol	up
F01_transcript_79126	down	Myristic acid	down
F01_transcript_79126	down	S-Adenosylmethionine	down
F01_transcript_79126	down	(E)-3-(4-Hydroxyphenyl)-2-propenal	up
F01_transcript_79126	down	Sinapoyl aldehyde	down
F01_transcript_79126	down	Kynurenic acid	down
F01_transcript_81509	up	beta-Sitosterol	up
F01_transcript_81509	up	9,10-DHOME	down
F01_transcript_88073	down	Glyoxylic acid	up
F01_transcript_88073	down	Sinapoyl aldehyde	down
F01_transcript_88073	down	5,7-Dihydroxyflavone	down
F01_transcript_88985	up	Glyoxylic acid	up
F01_transcript_88985	up	Kaempferide	up
F01_transcript_88985	up	5,7-Dihydroxyflavone	down
F01_transcript_88985	up	3-Hydroxybenzoic acid	up
F01_transcript_9341	up	beta-Sitosterol	up
F01_transcript_9341	up	S-Adenosylmethionine	down
F01_transcript_9341	up	5,7-Dihydroxyflavone	down
F01_transcript_9341	up	3-Hydroxybenzoic acid	up

F01_transcript_14491:*ASIL2*; F01_transcript_14819:*ASIL2*; F01_transcript_16804:*BZR1*; F01_transcript_17041:*BZR1*; F01_transcript_18056:*AS1*; F01_transcript_24248: *WRKY50*; F01_transcript_24692:*WRKY50*; F01_transcript_25025:*PTI5*; F01_transcript_31742:*PMA1*; F01_transcript_33244:*WER*; F01_transcript_34832: *RAP2-7*; F01_transcript_34879:*BHLH130*; F01_transcript_42053:*EIN3*; F01_transcript_43678: unknow; F01_transcript_48038:*WRKY70*; F01_transcript_48636: *SOC1*; F01_transcript_50082:*HY5*; F01_transcript_54031:*BZR1*; F01_transcript_57597:*MYB*; F01_transcript_58757:*LHY*; F01_transcript_65141:*LUX*; F01_transcript_67070: *BHLH130*; F01_transcript_68607: *MYB1R1*; F01_transcript_70090:*ERF038*; F01_transcript_79126:*BZIP43*; F01_transcript_81509:*APL*; F01_transcript_88073:*EIN3*; F01_transcript_88985:*BAM7*; F01_transcript_9341:*EIN3*

Table S7. List of RT-qPCR primers

ID	Sequence(5'-3')	Expected amplicon size（bp）
F01_transcript_42053	F:ACCATACACCACCTACATTCG<br>R: GCCCTCCAACACTATTCCT	158

F01_transcript_9341	F: ACGATCTCATGTCGCTCTA<br>R: GCAGTGTTGGCGTTGTA	190
F01_transcript_60137	F: TACCTCTGAACCCGTTGC<br>R: ACAGCCCTTGAATCTCCA	105
F01_transcript_40403	F: AACGAGCAGAGTTGTCCCT<br>R: AGCCCTTGAATCTCCACAG	130
F01_transcript_54031	F: GCTGCTCCCACTTCACCTA<br>R: ACCTTCCCATGCCTTCACT	156
F01_transcript_17041	F: ACACTGCGACAACAACGA<br>R: GTAAGAAGGTGCGGGACT	199
F01_transcript_33286	F: TCAAAGGGTTTCAAGTGCCA<br>R: CCTCACCAGCATCCTCATCT	108
F01_transcript_35805	F:CACAAGGGCATGGCAAGAG<br>R:GCAATGGCGGAAGTACGAG	111
F01_transcript_46007	F:TTATCGTCGTCGGTCTCG<br>R:AGGACTGCCAGAATGAACG	183
F01_transcript_54233	F: TGGCTACGACAACACCC<br>R: CGGCACAGAGTAATCCC	174
F01_transcript_14367	F: GGAAGGCATGGCCGATAAG<br>R: AGGCATTGTTTGGCTCACG	86
F01_transcript_63376	F: GTGGTGGAACGATCTCCG<br>R: TGTAGCCGCCATAAGGGT	170
F01_transcript_23979	F: CTGGACGAGAATCTGCTTT<br>R: GCTGCGGTGGTAATGTTT	150
F01_transcript_32117	F:AAGGCTGCTAAAGGGAGGA<br>R:CAGAAGGGCACATGAAAGA	139
F01_transcript_66084	F: GTCTTTCATGTGCCCTTCG<br>R: ATCGCCACCATCATCTACC	154
TIP41	F:TGGCTCAGAGTTGATGGAGTGCT<br>R:CTCTCCAGCAGCTTTCTCGGAGA	74
